# Supplementary material for: Joint representation of molecular networks from multiple species improves gene classification
Source: PLoS Comput Biol. 2024 Jan 10;20(1):e1011773. doi: 10.1371/journal.pcbi.1011773 (PMC10805316; doi:10.1371/journal.pcbi.1011773)
Supplement: S1 File — This information is organized under the following sections: multi-species networks, creating multi-species network representations, properties of gene set collections, optimal choices for gene classification using multi-species networks, statistical tests for main results, adding orthologs as positives, cross-species knowledge transfer, same-species prediction vs. full cross-species prediction, adding genes from multiple species during training, predicting model species counterparts of human diseases, and additional PCA plots. (PDF) [file pcbi.1011773.s003.pdf]

Joint representation of molecular networks from multiple species improves gene classification

S1 File

Multi-species networks

Table A shows the properties of networks from multiple species considered in this study, obtained from BioGRID [1] (v3.5.175) and IMP [2] (v2). Note that zebrafish was included only in some analyses in this work due to its small network size in BioGRID and limited number of known gene annotations to biological processes and phenotypes.

Table A: Network Coverage and Density. Genes: Number of genes in the network, Edges: Total number of edges in the network, Density: Ratio of number of edges / total possible edges

Species	BioGRID Number of Genes	BioGRID Number of Edges	BioGRID Density	IMP Number of Genes	IMP Number of Edges	IMP Density
human	17,692	338,669	0.0022	28,443	77,361,677	0.19
mouse	7,296	20,673	0.0008	36,242	250,547,482	0.38
zebrafish	208	216	0.01	22,646	64,559,106	0.25
fly	9,191	60,280	0.0014	20,397	112,978,222	0.54
worm	6,210	23,358	0.0012	21,030	55,513,529	0.25
yeast	6,144	537,204	0.0285	6,291	4,086,174	0.21

The first step in GenePlexusZoo is to connect gene networks from different species by adding an edge between pairs of genes if they belonged to the same EggNOG orthologous group [3]. For each species, **Fig A in S1 File** shows the number and fraction of genes in that species network that is part of a cross-species edge when combined with a network from another species.

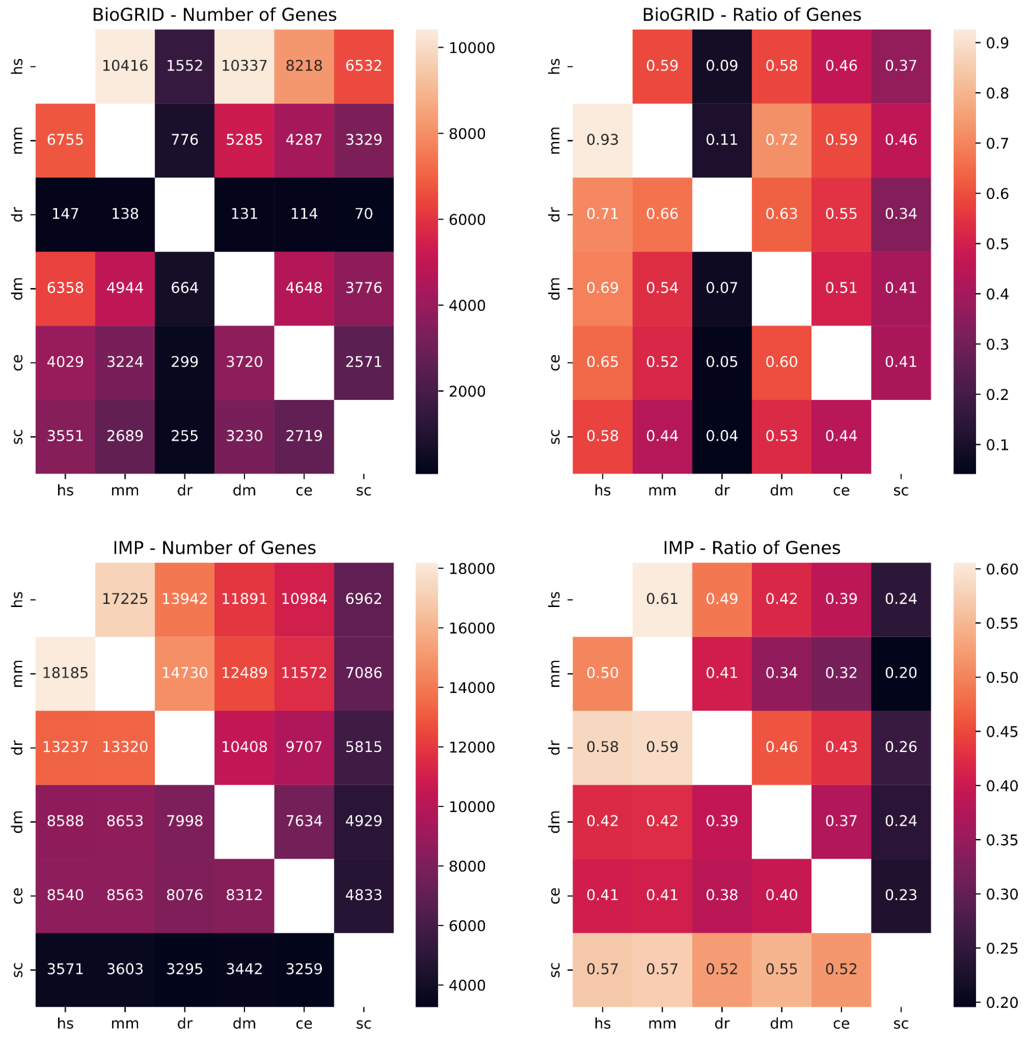

Fig A: Number and ratio of cross-species network connections. A row in each heat map in the left column shows the total number of genes in one species (row) present in a network of species combined with the network of another species (column). The corresponding entries in each heat map in the right column show the fraction of genes with a cross-species edge (when combined with a network from one other species). The top and bottom rows correspond to networks from BioGRID and IMP, respectively.

These cross-species gene links were weighted in two different ways (**Fig B in S1 File**; which were then compared). In the ‘uniform edge weighting strategy’, the edge weight was set to the same value for all cross-species edges. We devised a second ‘degree weighting strategy’ that weights edges differently based on the degrees of the incident gene nodes in their respective within-species networks. Specifically, for a pair of genes *u* and *v* in two different species, the cross-species edge weight, w_c, is given by:

$$w_c(u, v) = \frac{(C * W_{within})}{N_{orthos}}, \qquad \text{(eqn. S1)}$$

Here, W_{within} is the degree of the source node *u* when only considering within-species edges, N_{orthos} is the total number of ortholog connections for *u*, and *C* is a scaling parameter. The

parameter *C* influences the *node2vec* in how likely it is to generate walks that, after landing on node *u,* will next traverse to a node in the same species or to a node in a different species.

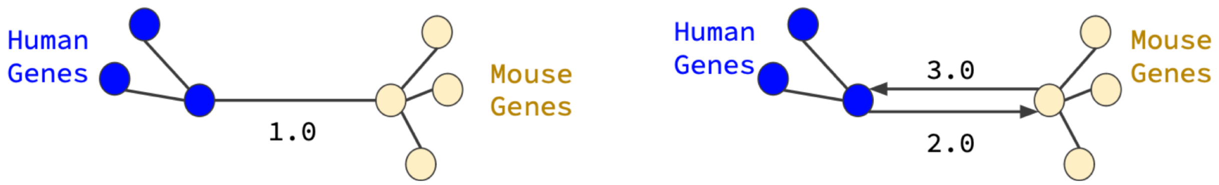

Fig B: Edge weighting strategies for connecting genes across species. The uniform edge weighting strategy sets all cross species edges to the same value (left). The degree weighting strategy (shown with C = 1) creates directed edges by considering both the within-species degree of the source node and the total number of cross-species edges the source node is incident on (right).

Creating multi-species network representations

After connecting networks from multiple species, we derived representations (to serve as features for ML) using four different methods:

1. **AdjMat**: Adjacency matrix representation of the networks where the features directly use the edge weights for all the connections. That is, each gene's feature vector is its corresponding row in the adjacency matrix.
2. **RWR**: The influence matrix representation generated with a random-walk-with-restart kernel [4].
    - The influence matrix $F = \beta[I - (1 - \beta)W_D]^{-1}$, where, β is the restart parameter, I is the identity matrix, and W_D is the degree-weighted adjacency matrix given by $W_D = AD^{-1}$, where $D \in R^{|V|x|V|}$ is a diagonal matrix of node degrees. We used either the influence matrix directly or its transpose.
3. **SVD**: Embedding of the adjacency matrix using SVD with or without column-wise standardization of the adjacency matrix before SVD.
4. **N2V**: Embedding of the adjacency matrix using *node2vec* [5].

Properties of gene set collections

This section reports the properties of the various gene set collections. The following information is contained in these tables (* denotes wildcard expression):

- **Task**: Name of the gene set collection; Gene Ontology (GO) [6], Monarch (MO) [7], and DisGeNet (DGN) [8,9].
- **Species**: Species for which the annotations of for; hs: human, mm: mouse, dr: zebrafish, dm: fly, ce: worm, sc: yeast
- **Network**: Network from which features are derived. Bio : BioGRID
- **NumSets**: Total number of sets that passed the threshold
- **All***: Considering all genes annotated to the set
- **Trn***: Considering only annotated genes in the training set

- **Tst***: Considering only genes in the testing set
- **Min**: The number of genes annotated to the gene set with the least amount of annotations
- **Max**: The number of genes annotated to the gene set with the greatest amount of annotations
- **Mean**: Average number of genes annotated to sets in the collection
- **Median**: Median value of the number of genes annotated to sets in the collection

Table B: Information on the non-redundant sets.

Task	Species	Network	NumSets	All-Min	All-Max	All-Mean	All-Median	Trn-Min	Trn-Max	Trn-Mean	Trn-Median	Tst-Min	Tst-Max	Tst-Mean	Tst-Median
GO	ce	Bio	58	24	148	75.9	70.5	12	122	59.7	57.5	10	44	16.2	14.5
GO	dm	Bio	133	23	188	88.7	87.0	12	174	71.3	71.0	10	77	17.5	15.0
GO	hs	Bio	201	21	100	62.1	64.0	10	87	45.5	46.0	10	51	16.6	14.0
GO	mm	Bio	76	21	78	45.7	44.5	10	64	31.8	31.5	10	29	13.9	12.0
GO	sc	Bio	113	20	198	84.0	75.0	10	182	60.9	52.0	10	83	23.1	17.0
MO	ce	Bio	52	33	147	87.2	86.5	23	123	70.7	71.0	10	41	16.5	15.5
MO	dm	Bio	3	149	185	169.7	175.0	139	173	158.0	162.0	10	13	11.7	12.0
MO	hs	Bio	208	20	99	60.0	57.5	10	87	45.8	43.0	10	30	14.1	13.0
MO	mm	Bio	82	22	70	41.1	40.5	11	59	28.6	27.0	10	20	12.5	12.0
MO	sc	Bio	50	30	196	108.4	101.0	11	172	87.1	78.0	10	97	21.3	14.5
DGN	hs	Bio	140	26	189	105.5	106.5	10	171	85.4	83.0	10	75	20.1	16.0
GO	ce	IMP	100	20	198	87.3	79.0	10	183	68.2	59.5	10	69	19.1	15.5
GO	dm	IMP	107	28	188	98.1	94.0	15	164	80.9	77.0	10	69	17.3	15.0
GO	dr	IMP	112	20	189	87.8	75.5	10	153	66.8	57.5	10	76	21.0	17.5
GO	hs	IMP	210	23	100	64.4	65.0	10	90	47.2	47.0	10	62	17.2	14.0
GO	mm	IMP	220	23	99	64.9	68.0	10	87	49.2	51.5	10	52	15.7	13.0
GO	sc	IMP	109	20	198	85.1	76.0	10	183	62.7	56.0	10	79	22.4	17.0
MO	ce	IMP	88	22	189	95.4	88.5	11	166	77.7	67.5	10	40	17.7	15.0
MO	dm	IMP	25	33	194	106.8	108.0	15	168	81.9	73.0	10	89	24.9	17.0
MO	dr	IMP	119	23	187	67.9	56.0	10	166	48.1	39.0	10	76	19.8	15.0
MO	hs	IMP	160	20	96	56.9	55.0	10	85	41.2	39.0	10	33	15.7	14.0
MO	mm	IMP	176	22	100	64.4	65.0	10	89	49.4	50.0	10	42	15.0	13.0
MO	sc	IMP	50	30	196	108.7	101.0	11	172	87.3	79.0	10	97	21.4	14.5
DGN	hs	IMP	143	22	199	107.4	106.0	10	179	87.5	82.0	10	74	19.9	16.0

Table C. Information on the matched non-redundant sets.

Task	Species	Features	Network	NumSets	All-Min	All-Max	All-Mean	All-Median	Trn-Min	Trn-Max	Trn-Mean	Trn-Median	Tst-Min	Tst-Max	Tst-Mean
GO	ce	ce_hs	Bio	16	21	126	70.6	69.5	9	100	56.6	56.0	10	26	14.0
GO	dm	dm_hs	Bio	66	17	188	78.0	65.0	7	166	61.5	47.5	10	58	16.4
GO	hs	ce_hs	Bio	16	29	192	113.9	120.5	16	164	89.8	95.0	10	47	24.1
GO	hs	dm_hs	Bio	66	38	190	124.5	131.0	12	168	97.1	99.0	10	82	27.4
GO	hs	hs_mm	Bio	80	19	195	129.4	137.5	8	176	106.3	113.5	10	48	23.1
GO	hs	hs_sc	Bio	92	16	190	99.8	95.0	6	170	72.1	67.5	10	83	27.7
GO	mm	hs_mm	Bio	80	18	141	66.0	64.5	6	116	51.6	47.0	10	35	14.3
GO	sc	hs_sc	Bio	92	15	170	64.5	52.5	5	151	43.6	34.5	10	74	21.0
GO	ce	ce_hs	IMP	30	20	163	68.6	57.0	9	134	53.1	41.5	10	56	15.6
GO	dm	dm_hs	IMP	53	20	197	84.1	67.0	7	177	67.7	51.0	10	69	16.4
GO	dr	dr_hs	IMP	24	20	187	84.4	68.5	8	142	62.4	46.5	10	45	22.0
GO	hs	ce_hs	IMP	30	32	199	130.9	150.5	15	168	98.7	109.5	12	89	32.2
GO	hs	dm_hs	IMP	53	20	196	120.4	125.0	5	163	90.1	86.0	10	89	30.3
GO	hs	dr_hs	IMP	24	37	197	117.3	107.0	13	174	93.8	79.5	10	71	23.5
GO	hs	hs_mm	IMP	198	20	198	122.9	129.0	5	179	97.0	98.0	10	87	25.8
GO	hs	hs_sc	IMP	87	16	196	105.3	102.0	6	175	76.0	76.0	10	89	29.2
GO	mm	hs_mm	IMP	198	17	200	88.4	81.0	5	180	70.3	62.0	10	62	18.1
GO	sc	hs_sc	IMP	87	17	170	65.4	50.0	7	154	44.7	35.0	10	73	20.8

Table D. Information on the full gene set collections.

Task	Species	Network	NumSets	All-Min	All-Max	All-Mean	All-Median
GO	ce	Bio	967	10	165	33.8	22
GO	dm	Bio	2002	10	195	41.7	27
GO	dr	Bio	96	10	35	14.3	13
GO	hs	Bio	3628	10	195	42.8	26
GO	mm	Bio	2794	10	145	33.0	23
GO	sc	Bio	1547	10	198	41.6	25
MO	ce	Bio	580	10	147	31.3	22
MO	dm	Bio	113	10	185	62.7	42
MO	dr	Bio	17	10	19	13.0	12
MO	hs	Bio	3112	10	196	38.2	23
MO	mm	Bio	2733	10	141	29.3	21
MO	sc	Bio	203	10	196	49.5	31
GO	ce	IMP	1289	10	198	39.5	23
GO	dm	IMP	2105	10	199	42.9	27
GO	dr	IMP	1205	10	196	40.4	24
GO	hs	IMP	3715	10	200	44.2	26
GO	mm	IMP	3755	10	200	42.3	26
GO	sc	IMP	1547	10	198	41.6	25
MO	ce	IMP	767	10	189	38.7	25
MO	dm	IMP	116	10	199	73.4	55
MO	dr	IMP	785	10	187	26.3	17
MO	hs	IMP	3236	10	199	39.0	24
MO	mm	IMP	3835	10	200	38.6	24
MO	sc	IMP	203	10	196	49.5	31

Optimal choices for gene classification using multi-species networks

To determine the optimal choice of network combination, representation, and hyperparameter setting that could improve the GenePlexus algorithm, we performed extensive evaluations on the tasks of predicting human and mouse annotations.

Which species to include?

The feature spaces were grouped into three main categories:

- **Single**: Features were only derived from network of the species the prediction task is in
- **Hs-mm**: Features were generated from jointly modeling human and mouse networks
- **All-species**: Features were generated from jointly modeling all species considered in this work (human, mouse, zebrafish, fly, worm, and yeast)

How to connect genes across species?

For Hs-mm and All-species, we weighted the edges for the cross-species connections based on two strategies (described in detail above):

- **Uniform**: All edge weights were set to the same value.
  - We evaluated weights equal to 1, 2, and 5.
- **Degree Weighted**: Directed edges were generated based on the within-species weighted-degree of the source node, the number of cross-species connections of the source node, and a scaling factor (see eqn. S1).
  - The scaling parameter was set to 1.0 or 0.50.

How to represent the network as features in the ML classifier?

Within each of the three main groups listed above, the features are generated using four different methods:

- **AdjMat**: There are no additional hyperparameters.
- **RWR**: We fixed the restart parameter to 0.8 and tested using either the influence matrix directly or its transpose.
- **SVD**: We tested two settings, one with and the other without standardizing the adjacency matrix column-wise before performing SVD.
- **N2V**: Involved parameters include *p* and *q*, which control how random walks are generated. We also performed some minimal tuning on the number of nodes per walk (*i.e.*, walk length), the number of walks per gene, and the number of epochs used when training the classifer.

For both SVD and N2V, we considered embedding dimension sizes of 500, 2000, and 10000. We note that, for the All-Species category, only the *node2vec* method could handle jointly modeling information from more than two species.

First, we observed that ‘degree-weighted’ connections between cross-species gene pairs (compared to ‘uniform’) resulted in more cohesion between genes in different species networks

(**Fig 6**; **Fig S–U in S1 File**). So, fixing those connections, we ran all combinations of network representation methods (and their parameter sets) and species choices for function and phenotype prediction tasks. **Fig C in S1 File** shows the results for the best hyperparameter from this analysis. This analysis required substantial computational resources and generated over 3.5TB worth of files just for human and mouse. For plots of all hyperparameter tuning results see the hyperparameter_tuning folder of the GenePlexusZoo-Manuscript github repository. Therefore, to compare across species other than just human and mouse, we picked one non-embedding method — AdjMat (over RWR) — and one embedding method — N2V with a p = 0.1, q = 0.001, dimension size of 2000, walk length = 120, number of walk = 8 and window size = 10 (over SVD) [**Fig D in S1 File**] — and used these two in all subsequent analyses (N2V Selected).

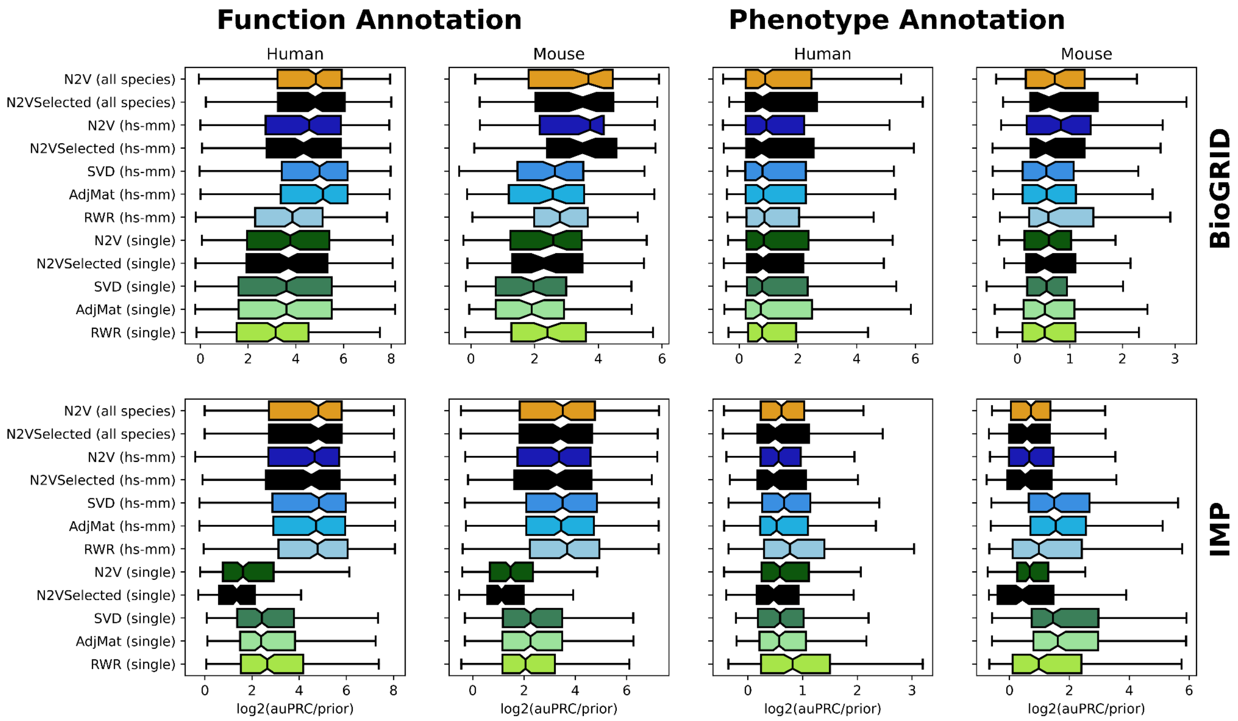

Fig C: Evaluation to determine optimal choices of settings and hyperparameters for improving GenePlexus with multi-species networks. Each group of boxplots shows the function and phenotype prediction performance for human and mouse using different species choice (for the network) and network representation methods (each using the best hyperparameter set, N2VSelected) based on networks from BioGRID and IMP.

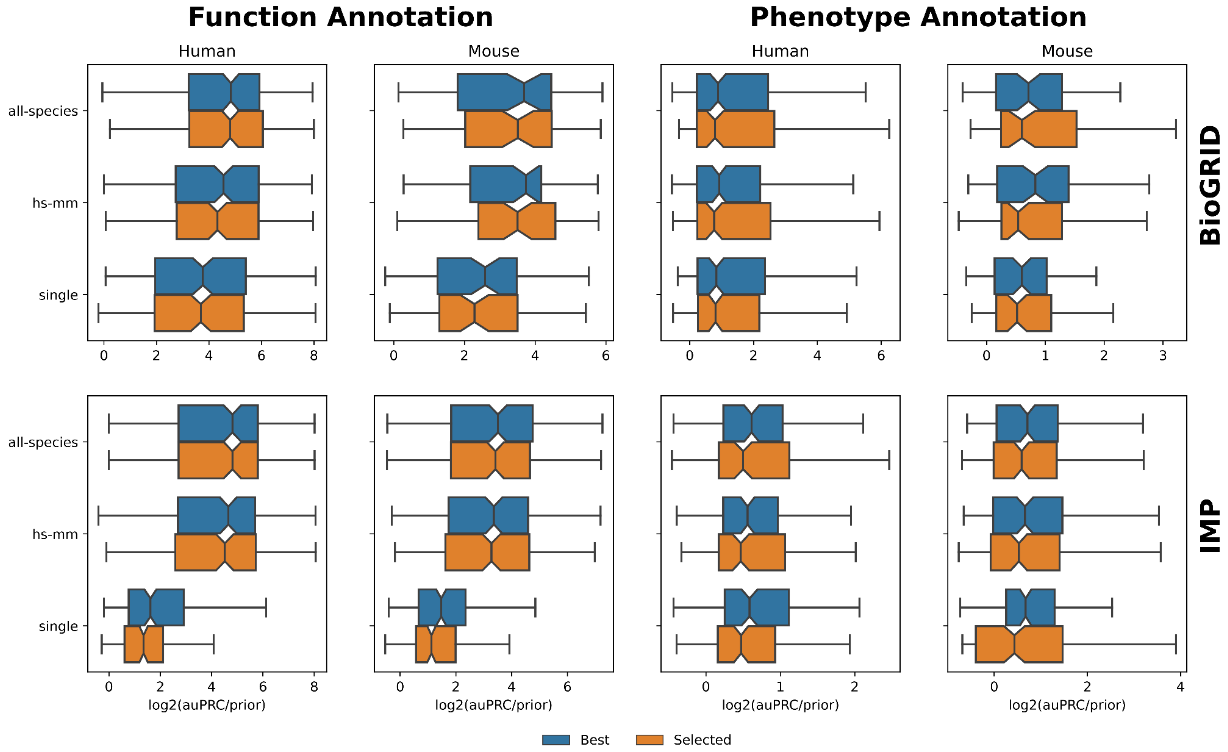

Fig D: Performance of the selected hyperparameter set for node embeddings. The boxplots show the log2(auPRC/prior) performance metric of the hyperparameter set we selected for N2V (orange) to use in the whole study compared to the performance of the best hyperparameter set (blue) across function and phenotype prediction tasks in human and mouse using networks from BioGRID and IMP.

Statistical tests for main results

To determine if a method was greater than the “baseline” method we performed a Wilcoxon signed-rank test as implemented in *scipy.stats.wilcoxon* between the *log2(auPRC/prior)* values for the two feature spaces. This test returns a p-value and a z-statistic, where the latter is a normalized version of the T-statistic. The p-value in the set of p-values generated for each feature set compared to the baseline is considered significant if the Bonferoni corrected p-value has a family-wise error rate less than 0.01.

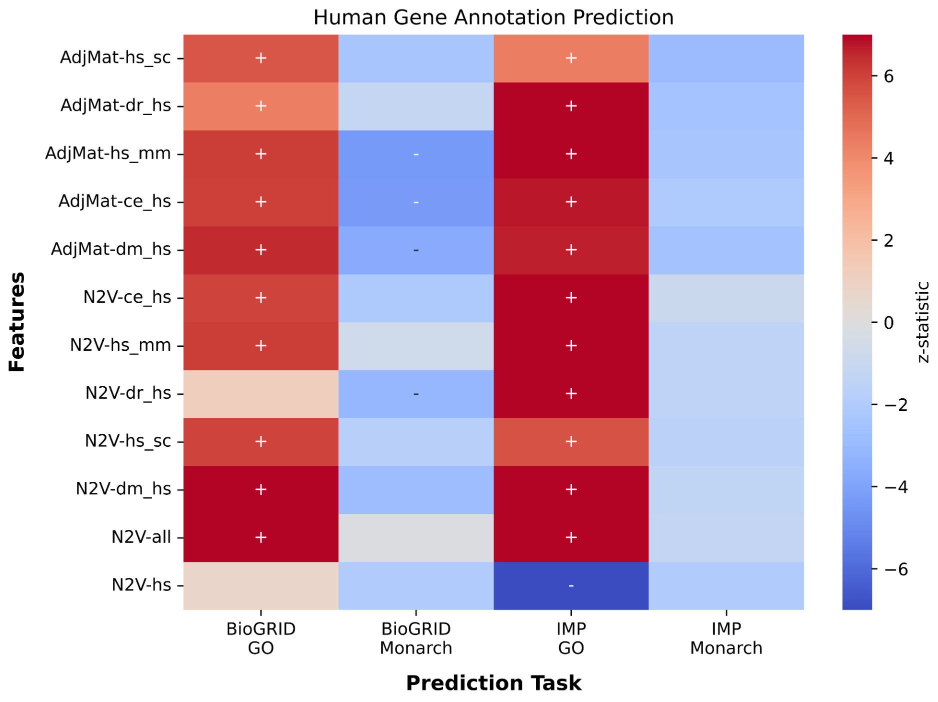

Fig E: Statistical tests for human gene annotation prediction. These results perform statistical tests for the results presented in **Fig 2**. The baseline method each method in a given prediction task is compared to is the adjacency matrix derived from only human network information (AdjMat-hs). Statistical significance is a FWER < 0.01 for a given prediction task. Higher values (red) of the z-statistic denote a representation outperforming the baseline ("+" denotes the difference being significant) and lower values (blue) of the z-statistic denote the baseline method having better performance ("-" denotes the difference being significant).

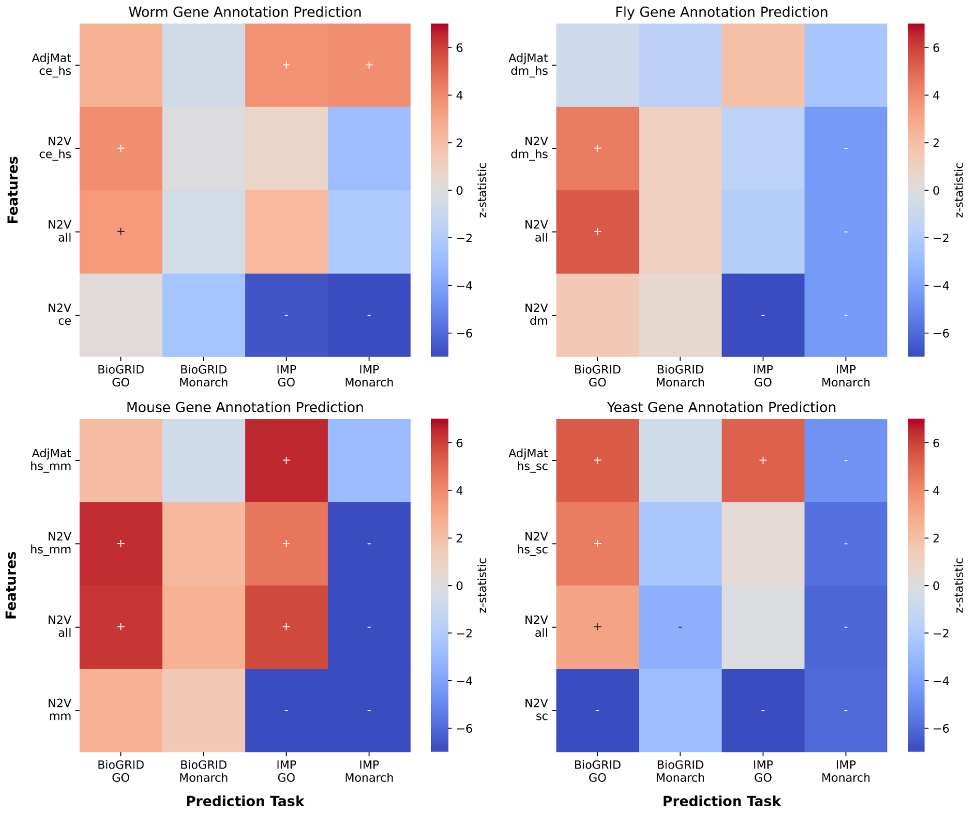

Fig F: Statistical tests for model species gene annotation prediction. These results perform statistical tests for the results presented in **Fig 3**. The baseline method each method in a given prediction task is compared to is the adjacency matrix derived from only model species network information for which the prediction task is for. Statistical significance is a FWER < 0.01 for a given prediction task. Higher values (red) of the z-statistic denotes a representation outperforming the baseline method (+ denotes the difference being significant) and lower values (blue) of the z-statistic denotes the baseline method having better performance (- denotes the difference being significant)

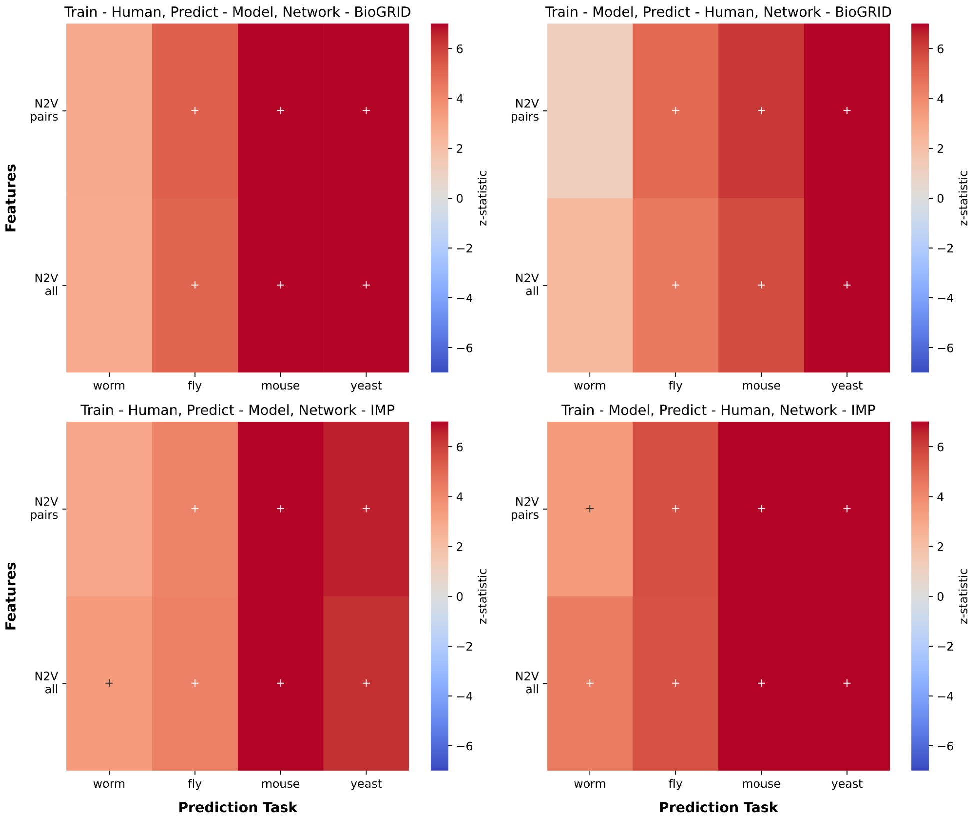

Fig G: Statistical tests for annotation prediction across species. These results perform statistical tests for the results presented in **Fig 4** and **Figs J-L**. The baseline method each method in a given prediction task is compared to is the adjacency matrix derived from network information of human plus the model species for which the prediction task is for. Statistical significance is a FWER < 0.01 for a given prediction task. Higher values (red) of the z-statistic denotes a representation outperforming the baseline method (+ denotes the difference being significant) and lower values (blue) of the z-statistic denotes the baseline method having better performance (- denotes the difference being significant)

Adding orthologs as positives

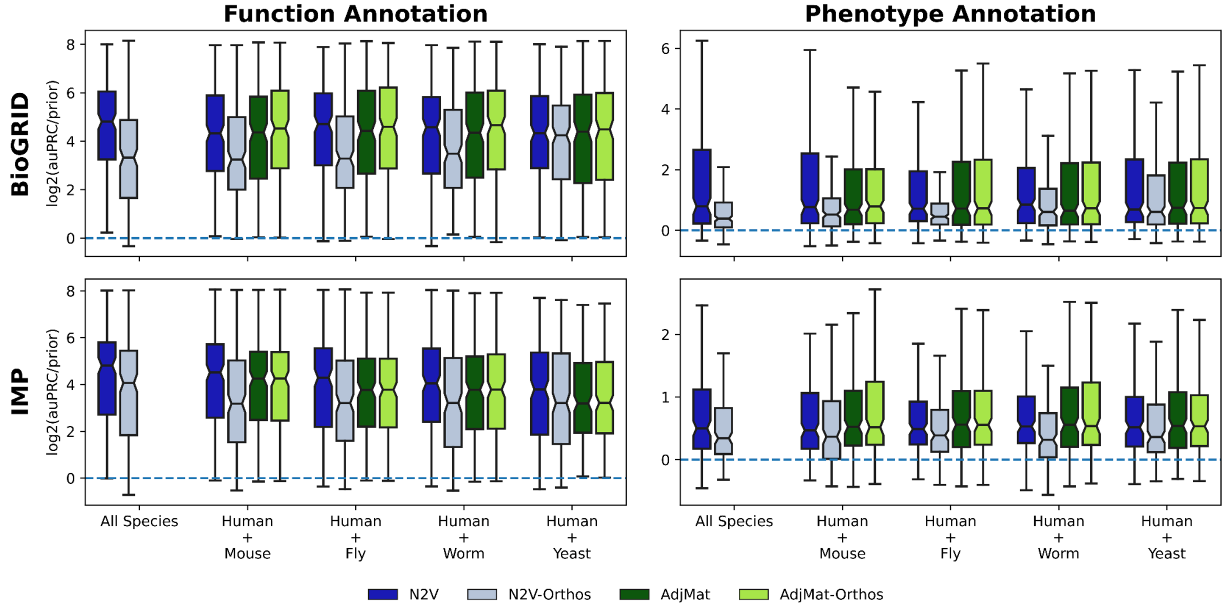

Fig H: Adding orthologs for predicting function- and phenotype-associated human genes. The same analysis as **Fig 2**, but now including orthologs from the model species as additional positive examples during training.

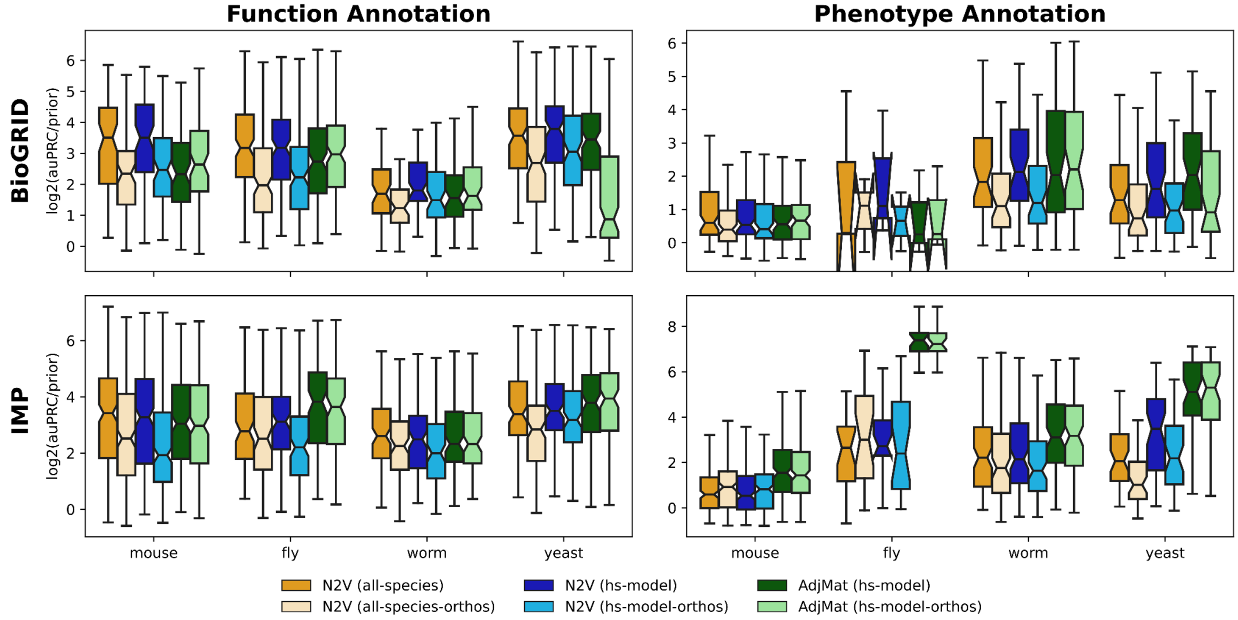

Fig I: Adding orthologs for predicting function- and phenotype-associated model species genes. The same analysis as **Fig 3**, but now including orthologs from the model species as additional positive examples during training.

Changing the ortholog database

We compared connecting genes across species together using two ortholog databases, eggNOG and WORMHOLE. eggNOG is a graph-based orthology method that more liberally assigns genes across species as ortholog pairs leading to a high number of genes with multiple orthologs, albeit at the expense of the percentage of gene pairs that are least diverged orthologs. WORMHOLE is a machine learning orthology method that integrates data from 17 different orthology databases. The ground truth labels for the classifier are least divergent ortholog pairs from the PANTHER database, thus WORMHOLE has far fewer ortholog pairs and percentage of genes with multiple orthologs compared to eggNOG, albeit with a much higher percentage of gene pairs that are least diverged orthologs.

The results show that models using eggNOG have better or equivalent performance than those using WORMHOLE, although not in all cases. Note that these evaluations were only carried out for the embedding methods as 1) PCA plots show that ortholog connections are much more important in embedding methods than adjacency matrix models, 2) hyperparameter tuning results show the adjacency matrix representations are not sensitive to the weighting scheme in which orthologs are connected and 3) the computational time to train models that use the adjacency matrix representation of species pairs is very intensive.

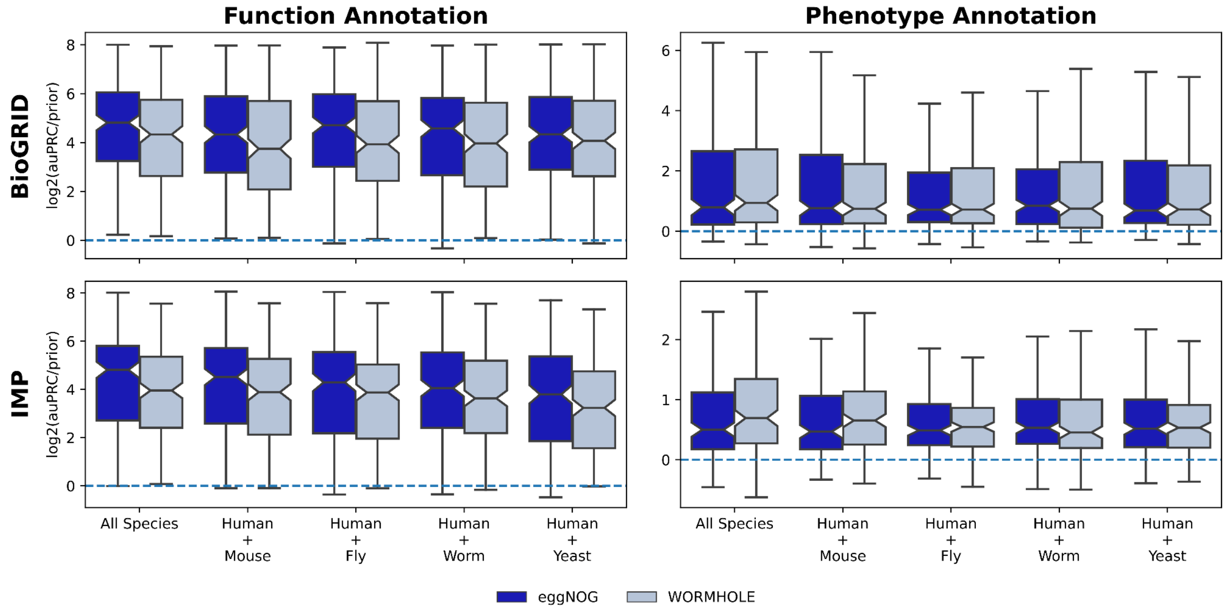

Fig J: Results for predicting function- and phenotype-associated human genes when changing the orthology database used to connect genes across species. The same analysis as **Fig 2**, but now looking at the difference in using the eggNOG orthology database versus WORMHOLE.

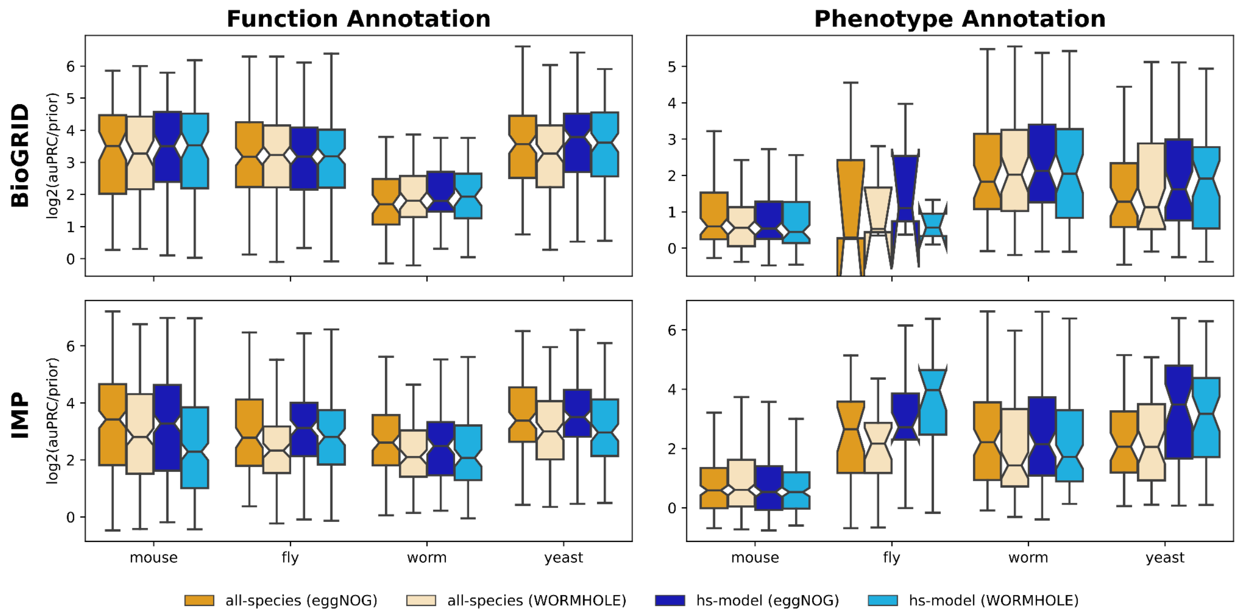

Fig K: Results for predicting function- and phenotype-associated model species genes when changing the orthology database used to connect genes across species. The same analysis as **Fig 3**, but now looking at the difference in using the eggNOG orthology database versus WORMHOLE.

Cross-species knowledge transfer

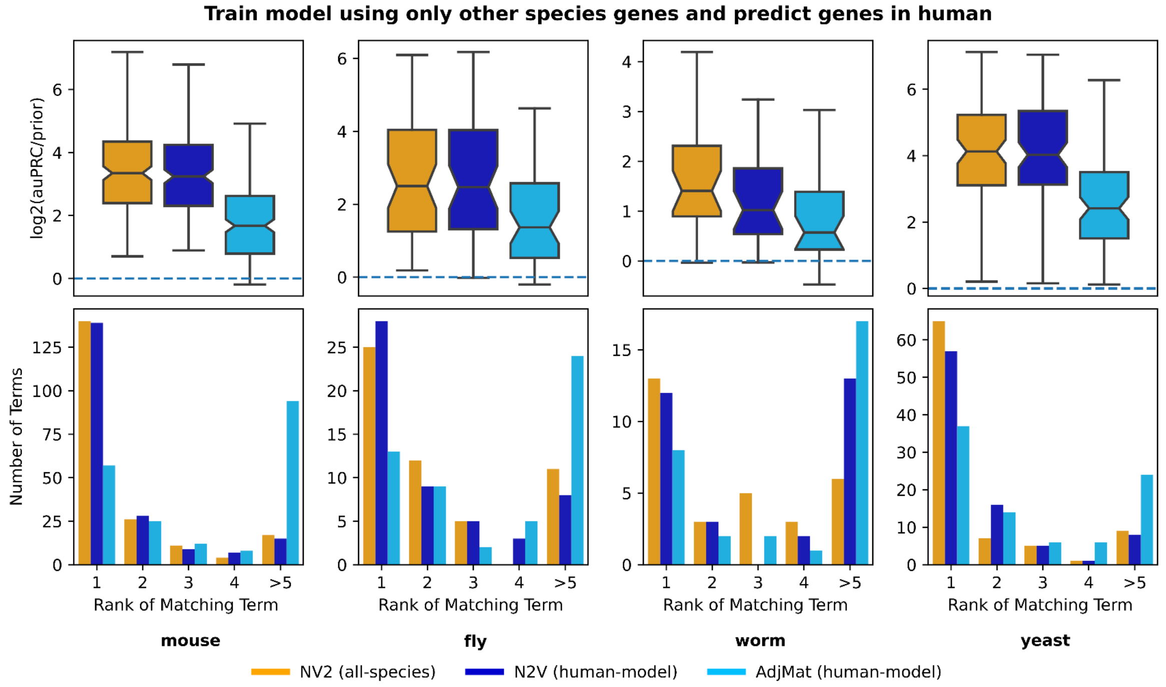

Fig L: Transferring knowledge from model species to human using human+model and all-species representations of networks from IMP. The boxplots (top row) show the $\log_2$(auPRC/prior) prediction performance of logistic regression classifiers that were trained using only model species genes annotated to a given biological process and then used to predict human genes annotated to the same biological

process. The barplots (bottom row) show the counts of where the log_2(auPRC/prior) for the matched biological process ranks in comparison to the other processes in the collection. Features for the classifier were created pairwise between human and a model species with IMP networks using the AdjMat (light blue) and N2V (dark blue) representations, as well as features from all species using the N2V (orange) representation.

The following two figures are the corresponding figures to **Fig 4** and **Fig G in S1 File**, respectively, except generated using the BioGRID network.

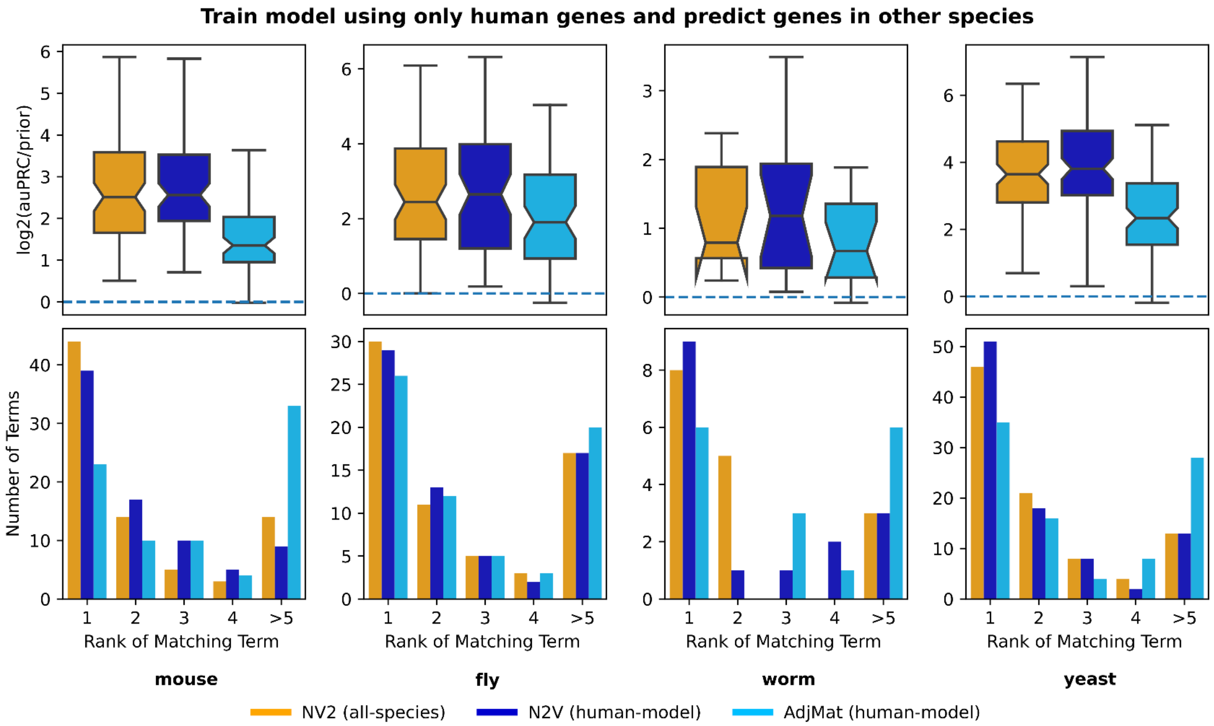

Fig M: Transferring knowledge from human to model species using human+model and all-species representations of networks from BioGRID. The boxplots (top row) show the $\log_2$(auPRC/prior) prediction performance of logistic regression classifiers that were trained using only human genes annotated to a given biological process and then used to predict model species genes annotated to the same biological process. The barplots (bottom row) show the counts of where the $\log_2$(auPRC/prior) for the matched biological process ranks in comparison to the other processes in the collection. Features for the classifier were created pairwise between human and a model species with BioGRID networks using the AdjMat (light blue) and N2V (dark blue) representations, as well as features from all species using the N2V (orange) representation.

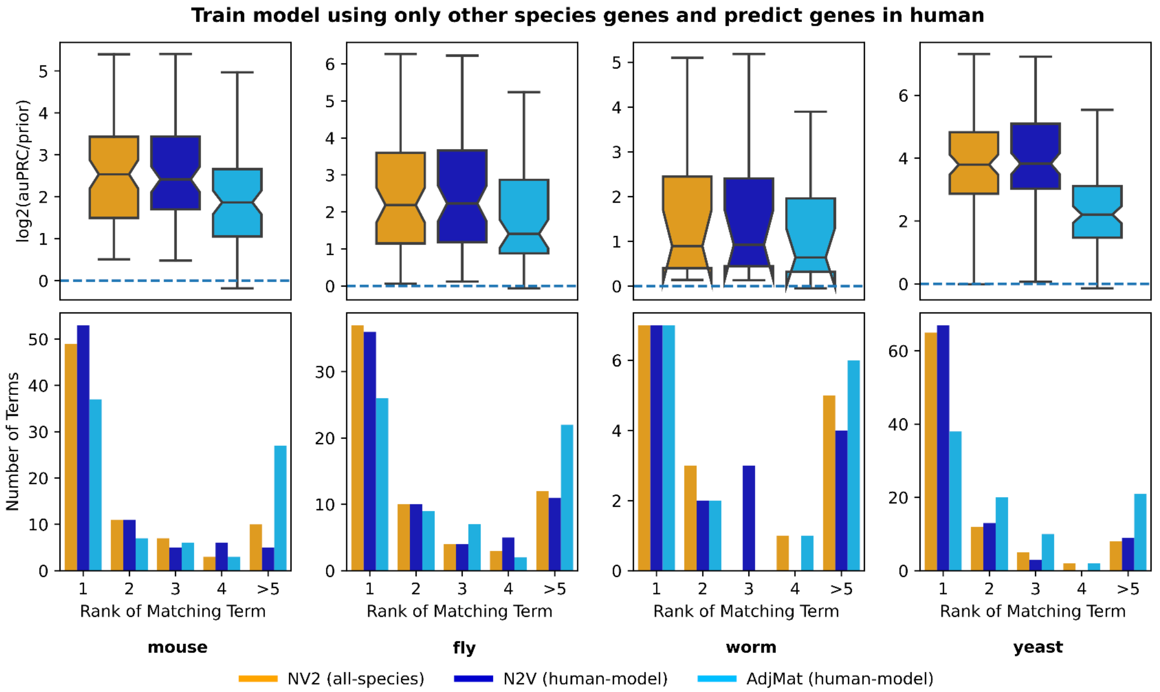

Fig N: Transferring knowledge from model species to human using human+model and all-species representations of networks from BioGRID. The boxplots (top row) show the $\log_2$(auPRC/prior) prediction performance of logistic regression classifiers that were trained using only model species genes annotated to a given biological process and then used to predict human genes annotated to the same biological process. The barplots (bottom row) show the counts of where the $\log_2$(auPRC/prior) for the matched biological process ranks in comparison to the other processes in the collection. Features for the classifier were created pairwise between human and a model species with BioGRID networks using the AdjMat (light blue) and N2V (dark blue) representations, as well as features from all species using the N2V (orange) representation.

Same-species prediction vs. full cross-species prediction

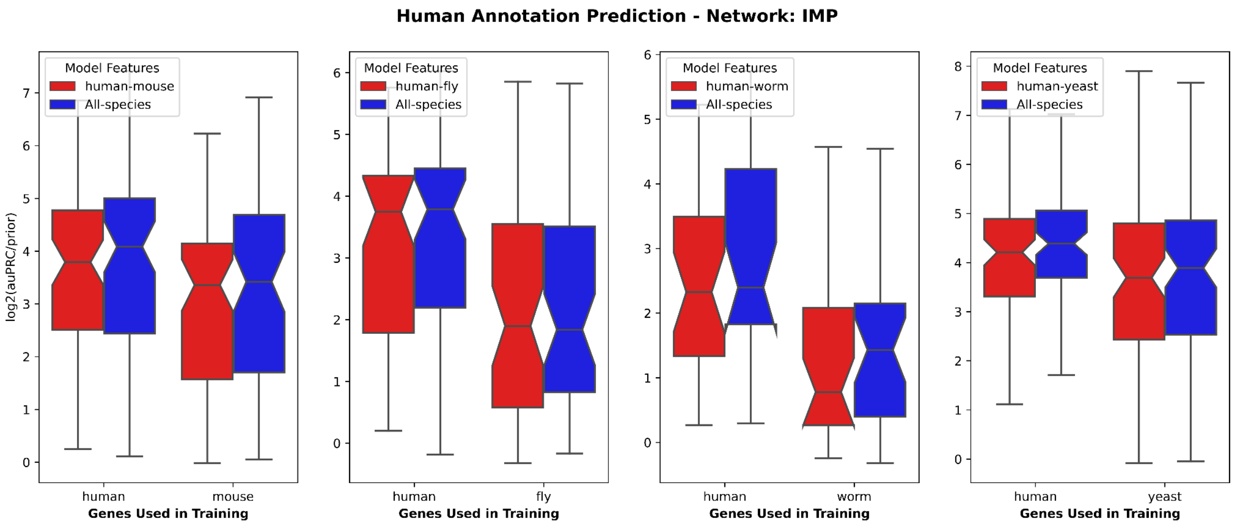

Fig O: Predicting human gene annotations by training classifiers with genes only from human or only from another species using IMP networks. Each panel corresponds to the analysis of human and one other model species. Within each panel, the boxplots show the performance on the task of predicting human genes annotated to biological processes using classifiers that were trained either on human genes only (left pair) or model species genes only (right pair), based on features from the human-model (red) or all six species (blue) networks from IMP.

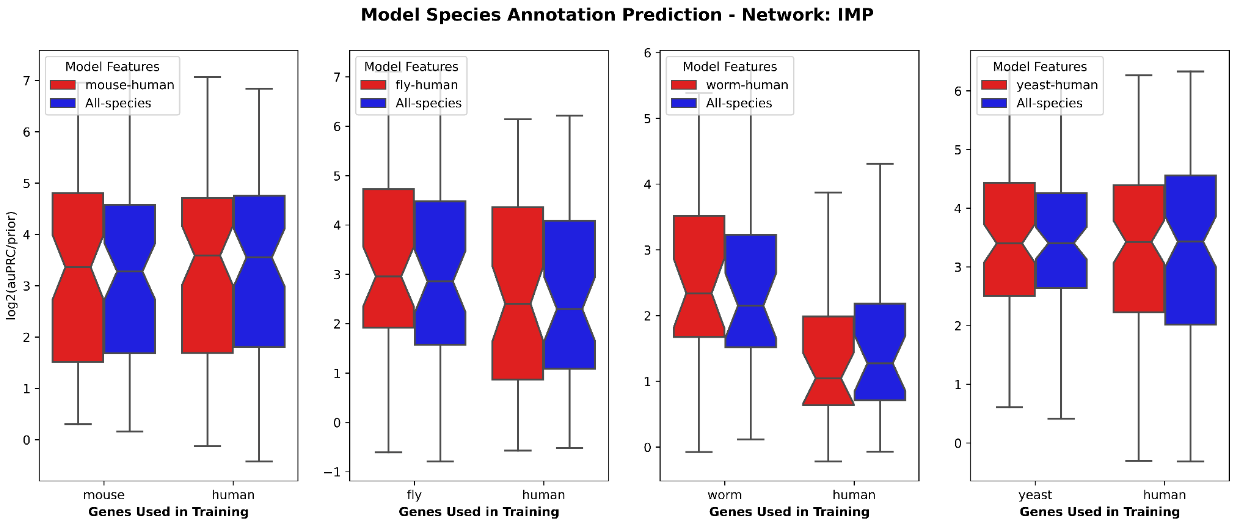

Fig P: Predicting model species gene annotations by training classifiers with genes only from that species or only from human using IMP networks. Each panel corresponds to the analysis of one model species with human. Within each panel, the boxplots show the performance on the task of predicting model species genes annotated to biological processes using classifiers that were trained either on model species genes only (left pair) or human genes only (right pair), based on features from the human-model (red) or all six species (blue) networks from IMP.

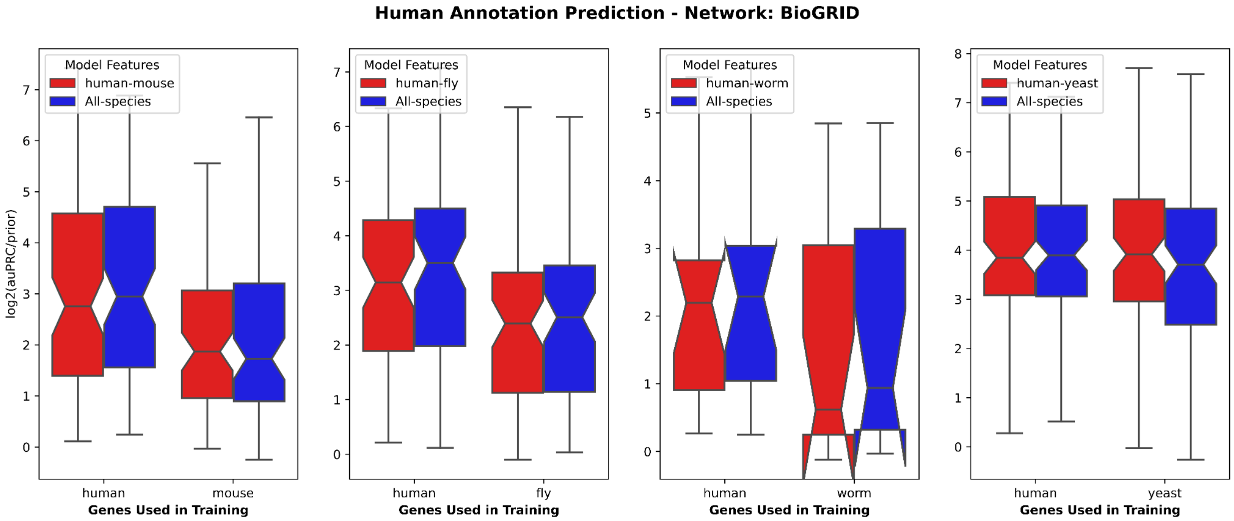

Fig Q: Predicting human gene annotations by training classifiers with genes only from human or only from another species using BioGRID networks. Each panel corresponds to the analysis of human and one other model species. Within each panel, the boxplots show the performance on the task of predicting human genes annotated to biological processes using classifiers that were trained either on human genes only (left pair) or model species genes only (right pair), based on features from the human-model (red) or all six species (blue) networks from BioGRID.

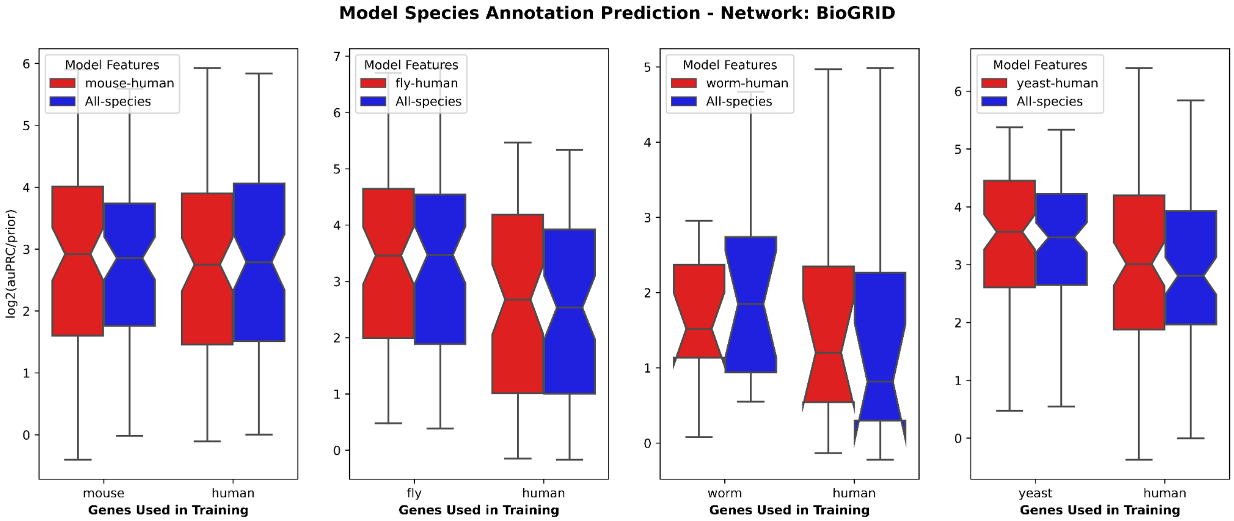

Fig R: Predicting model species gene annotations by training classifiers with genes only from that species or only from human using BioGRID networks. Each panel corresponds to the analysis of one model species with human. Within each panel, the boxplots show the performance on the task of predicting model species genes annotated to biological processes using classifiers that were trained either on model species genes only (left pair) or human genes only (right pair), based on features from the human-model (red) or all six species (blue) networks from BioGRID.

Adding genes from multiple species during training

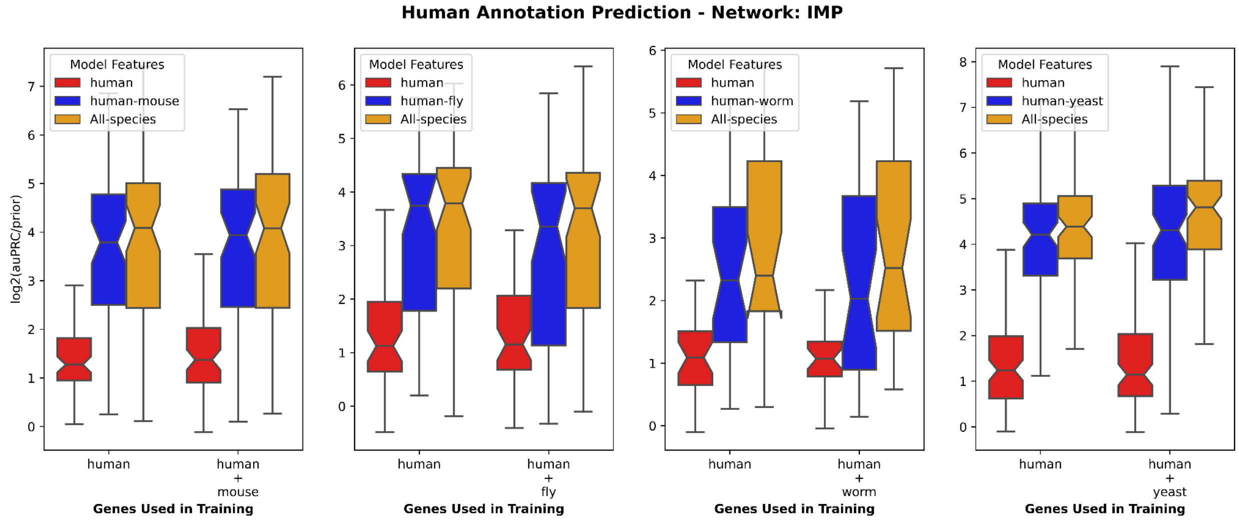

Fig S: Predicting human gene annotations by including genes from multiple species during training with IMP networks. Each panel corresponds to the analysis of human and one model species. Within each panel, the boxplots show the performance on the task of predicting human genes annotated to biological processes using classifiers that were trained either on human genes only (left pair) or human+model genes (right pair), based on features from the human (red), human+model (blue), or all six species (orange) networks from IMP.

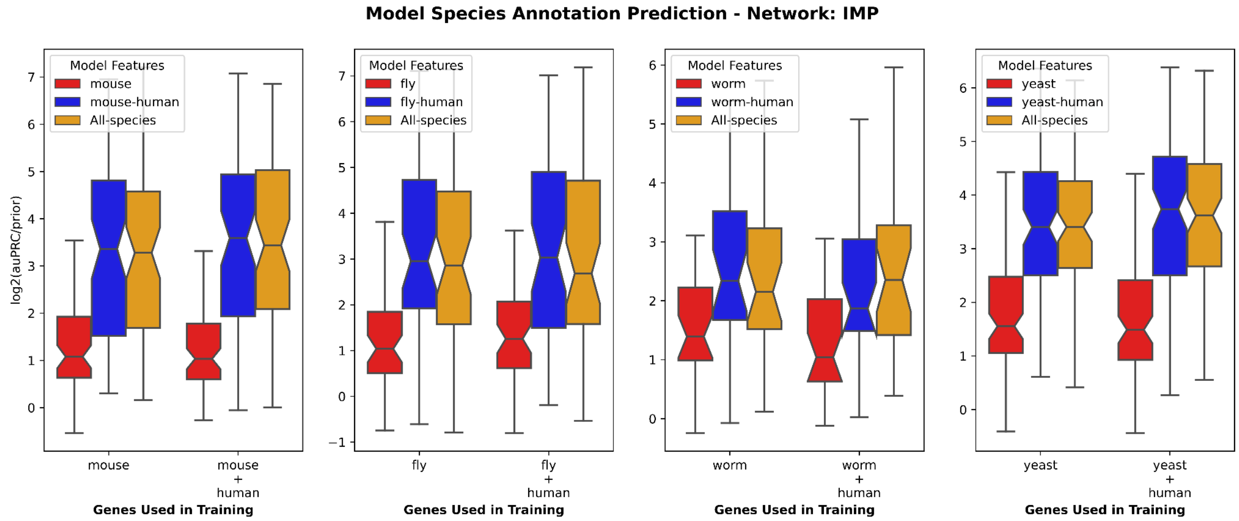

Fig T: Predicting model species gene annotations by including genes from multiple species during training with IMP networks. Each panel corresponds to the analysis of one model species with human. Within each panel, the boxplots show the performance on the task of predicting model species genes annotated to biological processes using classifiers that were trained either on model species genes only (left pair) or model+human genes (right pair), based on features from the model (red), model+human (blue), or all six species (orange) networks from IMP.

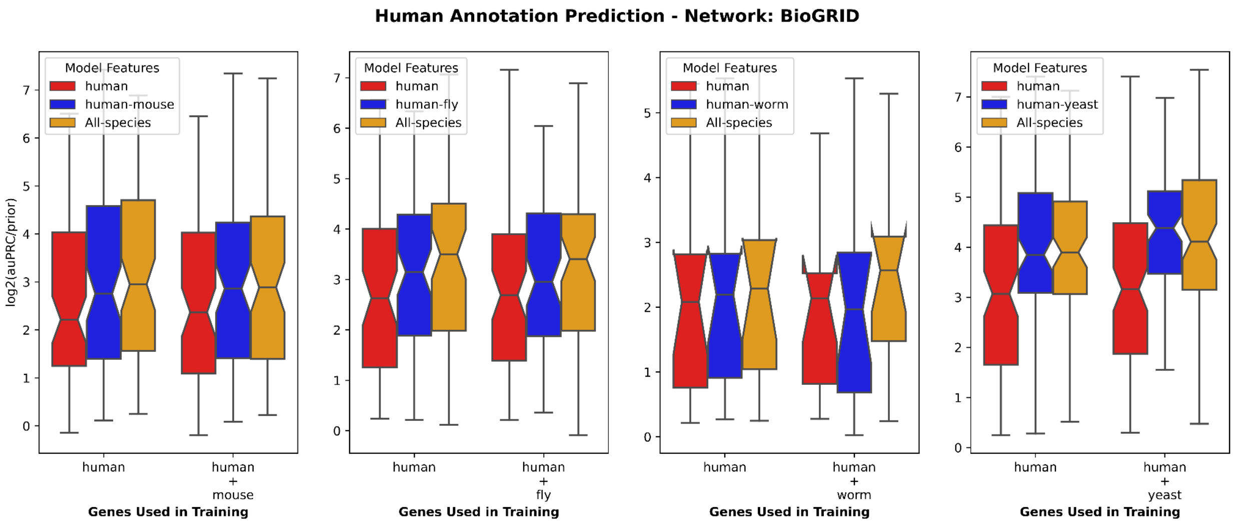

Fig U: Predicting human gene annotations by including genes from multiple species during training with BioGRID networks. Each panel corresponds to the analysis of human and one model species. Within each panel, the boxplots show the performance on the task of predicting human genes annotated to biological processes using classifiers that were trained either on human genes only (left pair) or human+model genes (right pair), based on features from the human (red), human+model (blue), or all six species (orange) networks from BioGRID.

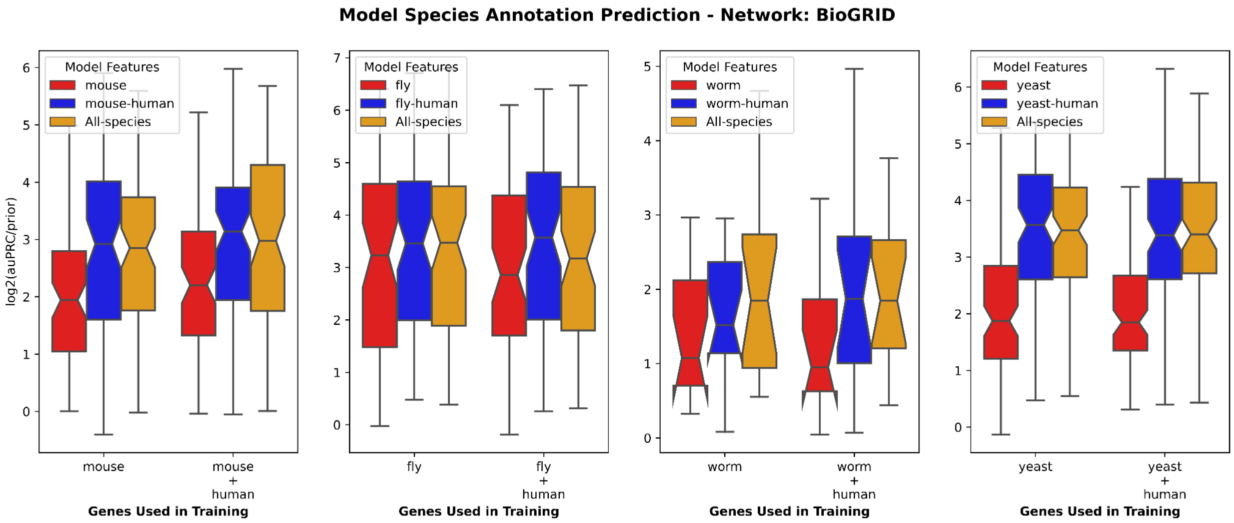

Fig V: Predicting model species gene annotations by including genes from multiple species during training with BioGRID networks. Each panel corresponds to the analysis of one model species with human. Within each panel, the boxplots show the performance on the task of predicting model species genes annotated to biological processes using classifiers that were trained either on model species genes only (left pair) or model+human genes (right pair), based on features from the model (red), model+human (blue), or all six species (orange) networks from BioGRID.

Predicting model species counterparts of human diseases

For a given species, the classifier trained on human disease gene annotations can be used to predict a probability of how associated every gene in all six species is to the human disease. To determine the z-score for the *i*-th gene, we use:

$$z_{gene,i} = p_i - \mu_p / \sigma_p \ , \qquad \text{(eqn. S2)}$$

where p_i is the probability of the *i*-th gene, μ_p and σ_p are the mean value and standard deviation of the probability distribution for a given species, respectively. To calculate the enrichment z-score for a GO or Monarch gene set, we use:

$$z_{set} = (\mu_{set} - \mu_p) / (\sigma_p / \sqrt{n}) \ , \qquad \text{(eqn. S3)}$$

where μ_{set} is the mean value of probabilities for only genes annotated to the set, n is the number of genes in the set, and μ_p and σ_p are the mean value and standard deviation of the probability distribution for a given species, respectively.

Table E: Bardet-Biedl Syndrome 1 top ten predicted genes in each species. Top ten genes in each species (columns) based on the z-score of the prediction probabilities from the classifier. Genes in each species are ranked independently, *i.e.*, genes from different species in the same row are not intended to correspond to each other.

Bardet-Biedl syndrome top ten associated genes					
human	mouse	fish	fly	worm	yeast
Bardet-Biedl syndrome 9	Bardet-Biedl syndrome 2 (human)	Bardet-Biedl syndrome 5	Meckel syndrome, type 1	Bardet-Biedl syndrome 7 protein homolog	chaperonin-containing T-complex subunit CCT7
Bardet-Biedl syndrome 7	Bardet-Biedl syndrome 5 (human)	nephronophthisis 1	Bardet-Biedl syndrome 5	Tetratricopeptide repeat protein 8	chaperonin-containing T-complex subunit CCT5
Bardet-Biedl syndrome 2	tetratricopeptide repeat domain 8	tetratricopeptide repeat domain 21B	Outer segment 2	Bardet-Biedl syndrome 2 protein homolog	1-phosphatidylinositol-3-phosphate 5-kinase
Bardet-Biedl syndrome 4	Bardet-Biedl syndrome 9 (human)	Bardet-Biedl syndrome 7	Bardet-Biedl syndrome 9	Protein pthb1 homolog	HSP70/90 family co-chaperone CNS1
tetratricopeptide repeat domain 8	centrosomal protein 290	intraflagellar transport 172	Bardet-Biedl syndrome 8	Nephrocystin-1-like protein	chaperonin-containing T-complex alpha subunit TCP1
Bardet-Biedl syndrome 5	intraflagellar transport 172	Bardet-Biedl syndrome 2	Bardet-Biedl syndrome 1	MecKel-Gruber Syndrome (MKS) homolog	Kar3p
tetratricopeptide repeat domain 21B	Bardet-Biedl syndrome 1 (human)	MKS transition zone complex subunit 1	Heat shock protein 60C	Bardet-Biedl syndrome 5 protein homolog	chaperonin-containing T-complex subunit CCT3
centrosomal protein 290	tetratricopeptide repeat domain 21B	centrosomal protein 290	Heat shock protein 60B	MecKel-Gruber Syndrome (MKS) homolog	chaperonin-containing T-complex subunit CCT2
Bardet-Biedl syndrome 1	MKS transition zone complex subunit 1	Bardet-Biedl syndrome 1	uncharacterized protein	Intraflagellar transport protein osm-1	lysophospholipase
leucine zipper transcription factor like 1	Bardet-Biedl syndrome 7 (human)	dynein, cytoplasmic 2, light intermediate chain 1	Intraflagellar transport 46	X-BoX promoter element regulated	transcription regulator CYC8

Table F: Bardet-Biedl Syndrome 1 top ten associated biological processes in each species. Top ten associated GO biological processes in each species (columns) based on the z-score of the predicted probability of the genes in each species. Processes in each species are ranked independently, *i.e.*, processes from different species in the same row are not intended to correspond to each other. Real relationships between these processes are presented in **Fig 5**.

Bardet-Biedl syndrome top ten associated biological processes					
human	mouse	fish	fly	worm	yeast
protein localization to cilium	cilium organization	melanosome transport	non-motile cilium assembly	cilium organization	protein folding
intraciliary transport involved in cilium assembly	non-motile cilium assembly	establishment of melanosome localization	cilium assembly	intraciliary transport	spindle assembly
intraciliary transport	cilium assembly	pigment granule transport	cilium organization	protein transport along microtubule	protein depolymerization
microtubule-based protein transport	regulation of cilium-dependent cell motility	melanosome localization	plasma membrane bounded cell projection assembly	microtubule-based protein transport	protein refolding
protein transport along microtubule	retina homeostasis	intracellular transport	cell projection assembly	cilium assembly	mitotic nuclear division
transport along microtubule	intraciliary transport	establishment of pigment granule localization	regulation of establishment of planar polarity	plasma membrane bounded cell projection assembly	mitotic spindle elongation
microtubule-based transport	striatum development	establishment of vesicle localization	axoneme assembly	cell projection assembly	spindle elongation
cytoskeleton-dependent intracellular transport	photoreceptor cell maintenance	pigment granule localization	microtubule bundle formation	protein-containing complex localization	glycerolipid metabolic process
microtubule-based movement	smoothened signaling pathway	vesicle localization	regulation of morphogenesis of an epithelium	microtubule-based transport	organelle transport along microtubule
ciliary basal body-plasma membrane docking	protein transport along microtubule	establishment of localization in cell	microtubule-based movement	transport along microtubule	transport along microtubule

Table G: Bardet-Biedl Syndrome 1 top ten associated phenotypes in each species. Top ten associated Monarch phenotypes in each species (columns) based on the z-score of the predicted probability of the genes in each species. Phenotypes in each species are ranked independently, *i.e.*, phenotypes from different species in the same row are not intended to correspond to each other.

Bardet-Biedl syndrome top ten associated phenotypes					
human	mouse	fish	fly	worm	yeast
Medial flaring of the eyebrow	photoreceptor inner segment degeneration	gastrulation disrupted, abnormal	abnormal planar polarity	ivermectin resistant	spindle morphology:abnormal
Hypoplasia of the ovary	absent sperm flagellum	notochord kinked, abnormal	lethal - all die during pharate adult stage	osmotic avoidance defective	chromosome segregation:abnormal
Postaxial hand polydactyly	Retinal degeneration	pronephric proximal convoluted tubule morphology, abnormal	sensory perception of sound process quality, abnormal	abnormal cilium morphology	nuclear position:abnormal
Hepatic fibrosis	retinal outer nuclear layer degeneration	pronephros development disrupted, abnormal	male semi-sterile	associative learning variant	vacuolar transport:decreased
Generalized hirsutism	enlarged third ventricle	somite increased width, abnormal	majority die during pharate adult stage	dye filling defect	vacuolar transport:abnormal
Multicystic kidney dysplasia	absent embryonic cilia	notochord increased width, abnormal	some die during first instar larval stage	vulva location variant	chemical compound accumulation:absent
Nephrotic syndrome	disorganized photoreceptor outer segment	convergent extension process quality, abnormal	cytokinesis process quality, abnormal	male response to contact defective	protein secretion:decreased
Polydactyly	abnormal renal tubule epithelial cell primary cilium morphology	pronephros cystic, abnormal	semi-fertile	ammonium chloride chemotaxis defective	cytoskeleton morphology:abnormal
Pigmentary retinopathy	small hippocampus	pronephric duct dilated, abnormal	sensory perception of touch process quality, abnormal	cephalic sensillum morphology variant	freeze-thaw resistance:decreased
External genital hypoplasia	abnormal motile primary cilium morphology	cilium Kupffer's vesicle decreased length, abnormal	photoperiod response variant	male mating efficiency reduced	protein activity:absent

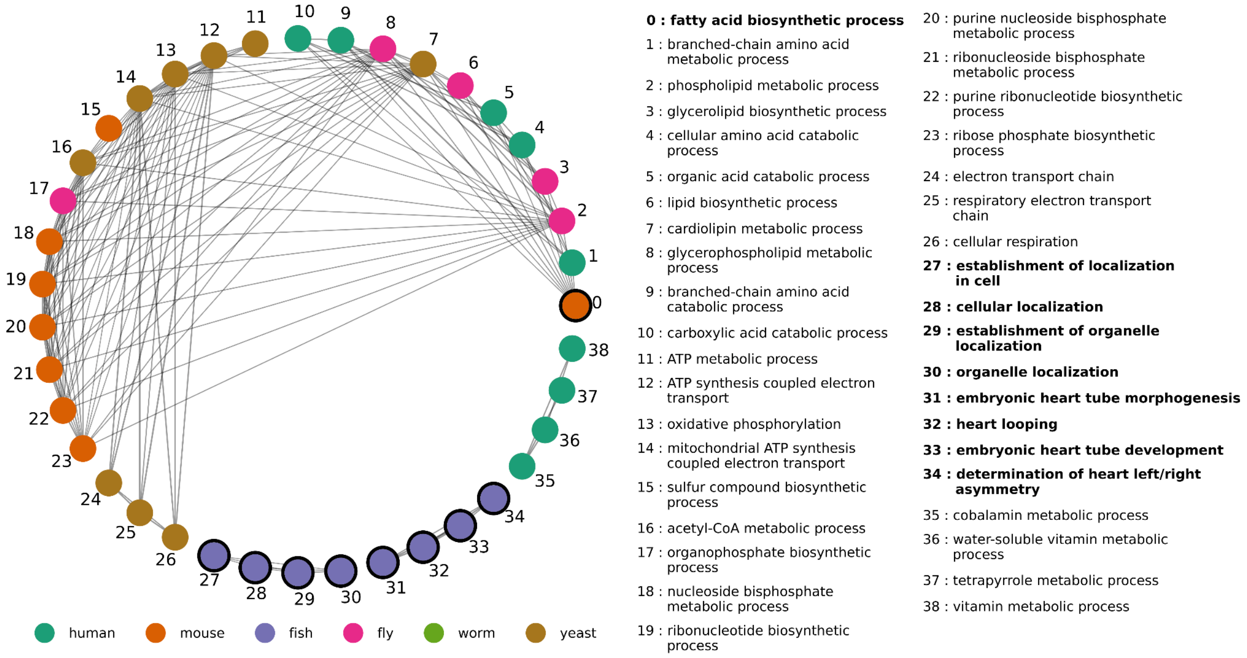

Fig W: The ten most enriched biological processes associated with the top genes in each species predicted to be related to organic acidemia (OA). A classifier trained using human OA genes and all-species N2V features was used to predict OA-related genes in the five model species. The figure shows the ten most enriched biological processes associated with the top-ranked genes in each species. Nodes represent biological processes and are colored by which species they were identified in. Edges represent pairs of semantically similar processes (scaled Resnik similarity based on the Gene Ontology). Isolated nodes are not shown. Nodes with thick borders represent biological processes where, in at least one species, none of the annotated genes are orthologous to any human OA gene.

Additional PCA plots

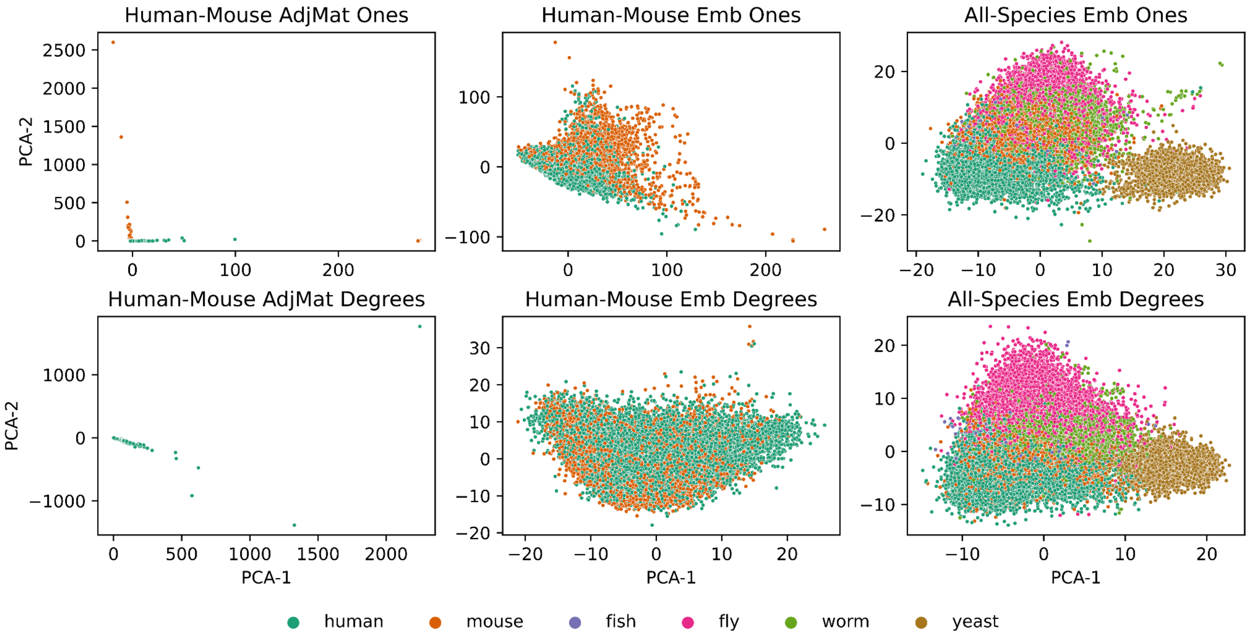

Fig X: PCA of adjacency matrix and node embedding representations of human-mouse and all-species networks. Each PCA plot shows the top two PCA components from the human-mouse adjacency matrix (first column), the human-mouse node embedding (second column) and the all species node embedding (third column) representations. The cross-species edge weights were created by always using a uniform edge weight with a value of one (top row) or by using degree based edge weights (bottom row) for the BioGRID network.

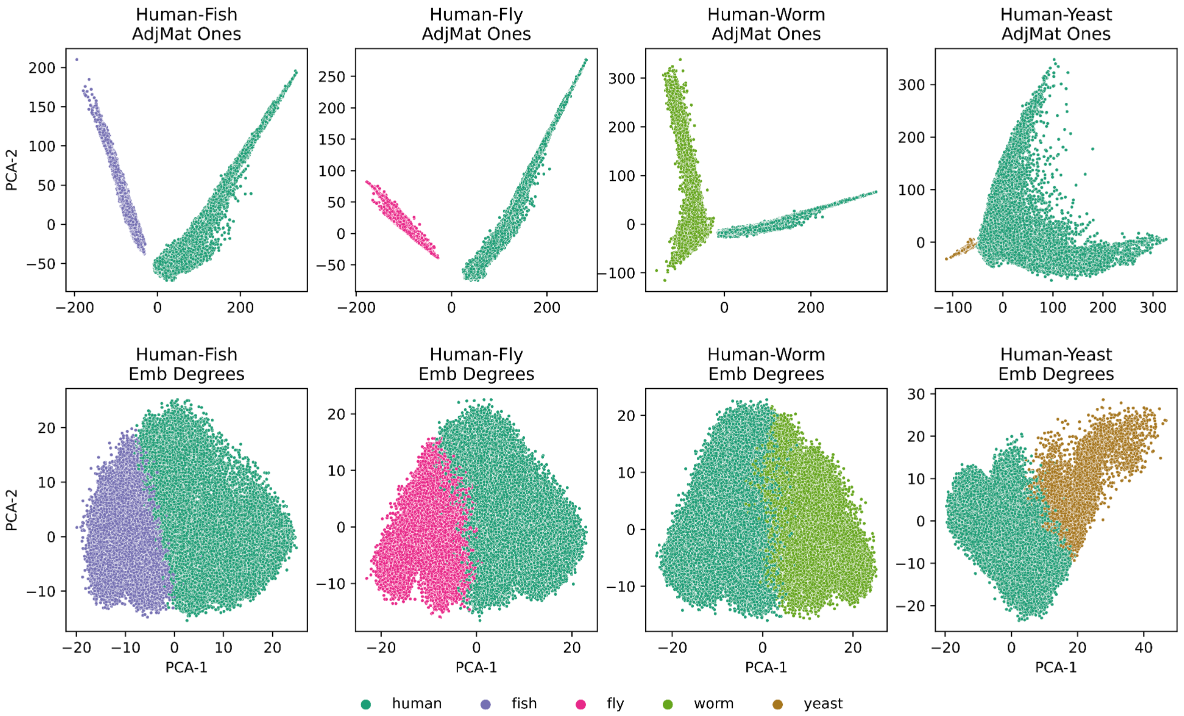

Fig Y: PCA of adjacency matrix and node embedding representations of human-model networks from IMP. Each PCA plot shows the top two PCA components from the adjacency matrix (using the uniform weighting strategy; top row) and node embeddings (using the degree based weighting strategy; bottom row) representations for the human network combined pairwise with fish, fly, worm, yeast in IMP.

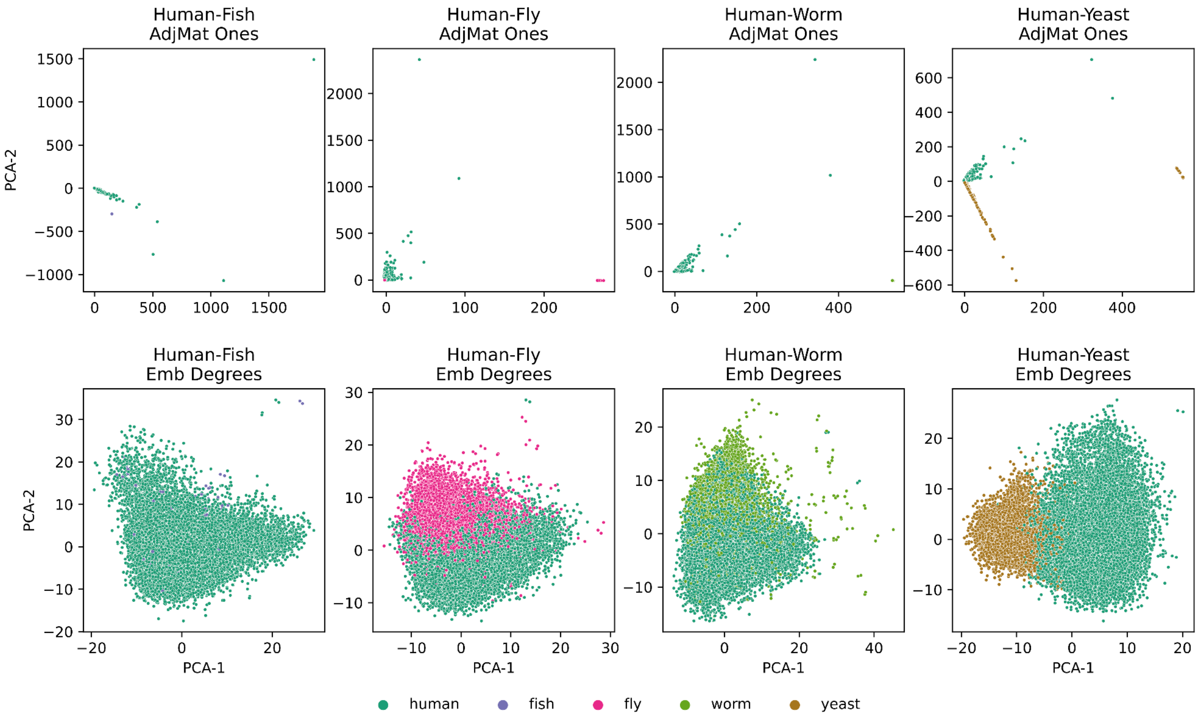

Fig Z: PCA of adjacency matrix and node embedding representations of human-model networks from BioGRID. Each PCA plot shows the top two PCA components from the adjacency matrix (using the uniform weighting strategy; top row) and node embeddings (using the degree based weighting strategy; bottom row) representations for the human network combined pairwise with fish, fly, worm, yeast in BioGRID.

References

1. Stark C, Breitkreutz B-J, Reguly T, Boucher L, Breitkreutz A, Tyers M. BioGRID: a general repository for interaction datasets. Nucleic Acids Res. 2006;34: D535–D539. doi:10.1093/nar/gkj109
2. Wong AK, Krishnan A, Yao V, Tadych A, Troyanskaya OG. IMP 2.0: a multi-species functional genomics portal for integration, visualization and prediction of protein functions and networks. Nucleic Acids Res. 2015;43: W128–W133. doi:10.1093/nar/gkv486
3. Huerta-Cepas J, Szklarczyk D, Heller D, Hernández-Plaza A, Forslund SK, Cook H, et al. eggNOG 5.0: a hierarchical, functionally and phylogenetically annotated orthology resource based on 5090 organisms and 2502 viruses. Nucleic Acids Res. 2019;47: D309–D314. doi:10.1093/nar/gky1085
4. Leiserson MDM, Vandin F, Wu H-T, Dobson JR, Eldridge JV, Thomas JL, et al. Pan-Cancer Network Analysis Identifies Combinations of Rare Somatic Mutations across Pathways and Protein Complexes. Nat Genet. 2015;47: 106–114. doi:10.1038/ng.3168
5. Grover A, Leskovec J. node2vec: Scalable Feature Learning for Networks. Proceedings of the 22nd ACM SIGKDD International Conference on Knowledge Discovery and Data Mining - KDD ’16. San Francisco, California, USA: ACM Press; 2016. pp. 855–864. doi:10.1145/2939672.2939754
6. Ashburner M, Ball CA, Blake JA, Botstein D, Butler H, Cherry JM, et al. Gene ontology: tool for the unification of biology. The Gene Ontology Consortium. Nat Genet. 2000;25: 25–29. doi:10.1038/75556
7. Mungall CJ, McMurry JA, Köhler S, Balhoff JP, Borromeo C, Brush M, et al. The Monarch Initiative: an integrative data and analytic platform connecting phenotypes to genotypes across species. Nucleic Acids Res. 2017;45: D712–D722. doi:10.1093/nar/gkw1128
8. Piñero J, Bravo À, Queralt-Rosinach N, Gutiérrez-Sacristán A, Deu-Pons J, Centeno E, et al. DisGeNET: a comprehensive platform integrating information on human disease-associated genes and variants. Nucleic Acids Res. 2017;45: D833–D839. doi:10.1093/nar/gkw943
9. Schriml LM, Mitraka E, Munro J, Tauber B, Schor M, Nickle L, et al. Human Disease Ontology 2018 update: classification, content and workflow expansion. Nucleic Acids Res. 2019;47: D955–D962. doi:10.1093/nar/gky1032
