# Supplementary material for: Joint representation of molecular networks from multiple species improves gene classification
Source: PLoS Comput Biol. 2024 Jan 10;20(1):e1011773. doi: 10.1371/journal.pcbi.1011773 (PMC10805316; doi:10.1371/journal.pcbi.1011773)
Supplement: Attachment — Submitted filename: GenePlexusZoo_PLoSCB-Response-to-Reviewers.pdf [file pcbi.1011773.s004.pdf]

Response to the Reviewers for PCOMPBIOL-D-23-01096
We would like to thank the reviewers for carefully reading our manuscript and for their helpful comments. We have provided detailed responses to all the comments below.

In the following, **Reviewer comments** are in **black**, **our responses** are in **blue**, and the **changes made to the text of our paper** are shown in **red**.

Response To Reviewer 1:
Comment 1: This is a very good article and can be accepted in its present form.
Congratulations to all the authors for a good study.

We thank the reviewer for their kind words and their recommendation for accepting the study as is.

Response To Reviewer 2:
Comment 1: The abstract outlines the development of "GenePlexusZoo," a framework for integrating molecular networks from multiple species into one feature space for network-based ML. It suggests this multi-species approach can enhance gene classification and knowledge transfer across species, even when inter-species correspondence is less evident based on orthologous genes.
The focus on a multi-species network representation and the emphasis on reusability for various types of prediction could be useful for researchers working on molecular network analyses and gene classification. The context is well established, stating the current limitations of single-species network analyses and the need for a unified approach.
The introduction establishes the importance of molecular interaction networks and highlights the challenges of single-species network analyses. It further emphasizes the value of cross-species knowledge transfer, especially for improving clinical trial outcomes. The paper places its claims within the context of existing literature, citing numerous studies that discuss the potential and pitfalls of current approaches. The identification of gaps in current methods namely an inability to handle multiple species networks, lack of incorporation of many-to-many orthology, and reusability across various tasks leads to the proposed solution, "GenePlexusZoo."
There are three key results that are mentioned in the Introduction and revisited later in the Results section:
Multi-species classifiers used on a single target species outperform classifiers trained on a single species.
The embedding methods introduced improve cross-species knowledge transfer for classification.
An example with a human disease gene-trained classifier being used to predict genes in other organisms.

We appreciate the reviewer's summary of the manuscript.

Comment 2: The methods section begins with network selection by describing multi-species networks from two sources (one sparse - BioGRID, one dense - IMP). It continues to describe

network connection with two edge weighting methods. Feature extraction is discussed next, primarily with the adjacency matrix and node2vec. There are several other methods outlined in S3 and they should be mentioned here as well (RWR, SVD).

We now mention RWR and SVD in the "Creating feature representations" section of the **Methods** section.

Comment 3: Gene set collection is divided into gene function prediction and phenotype prediction. For evaluation, the study-bias holdout scheme is innovative. It closely aligns with real-world challenges where novel genes are being discovered and existing genes are being better characterized. It's not clear if random training and testing was attempted or not from the text. A comment should be added here on whether or not standard splitting methods were tested.

We now mention how, in our previous work (Liu et al., 2020), we investigated three evaluation schemes — study-bias holdout, 5-fold CV (*i.e.*, randomized training/testing), and temporal holdout — and discussed the differences in that work. However, as many of the cross-species classifiers take significantly more computational resources (time and memory), in this study, we restrict ourselves to just study-bias holdout. Text has been added to the "Evaluation scheme" section in the **Methods** section to clarify this choice.

Comment 4: The gene classifier makes use of a standard logistic regression with L2 regularization supervised-learning approach, backed by previous studies. This is a simple yet powerful method for classification tasks but it doesn't look like any other classifiers or regularization methods were evaluated. There should be some more justification in the text for the single method or at least an acknowledgment of future research avenues to improve performance with different classifiers.

We have extensively benchmarked the GenePlexus method using a variety of learning algorithms. In particular, our first GenePlexus work (Liu et al., 2020) describes comparisons between logistic regression with L1 or L2 regularization, support vector machines with linear, radial basis function, and polynomial kernels, as well as a random forest classifier. In more recent work (Liu et al., 2023), we compared using logistic regression and graph neural networks, showing that logistic regression outperformed these deep learning models, most likely due to the limited number of positively labeled samples for most specific biological functions. Text has been added to the "Network-based gene classifiers" section in the **Methods** section to reference this.

Comment 5: The evaluation metric of choice is auPRC and is selected over auROC due to the imbalanced dataset (making it more suitable for gene classification tasks). I have a couple of small issues with this section. The machine learning terminology may not be immediately understood by readers so precision and recall should be defined to make it instantly understandable to people familiar with other error rate terminology.

In the first section of the "Evaluation metrics" section, we have now added more text that clearly explains how the log2(auPRC/prior) metric is calculated. The third paragraph of the same section also explains our reasons for using auPRC as opposed to auROC.

Comment 6: Also, the explanation of auPRC and log2 needs to explicitly state what is increasing 2-fold over random chance.

We agree that such a clarification will be helpful. For auPRC, the value of a random classifier depends on the relative proportion of positives to negatives. For example, in a label set with 100 positives and 900 negatives, a random classifier expected auPRC would be a value of 0.1 (which is its prior = #P / (#P + #N)). Thus, the log2(auPRC/prior) metric quantifies the 2-fold increase of the auPRC of the real classifier over that of a random classifier. We have added text to the second paragraph of the "Evaluation metrics" section with this interpretation of the metric.

Comment 7: Additionally, in the next paragraph, it is not clear what the stated goal is in terms of error control. Saying 'top predictions are as correct as possible' is too vague and should be correctly defined.

We agree that the language here is vague and needs improvement. We have added text to the third paragraph of the "Evaluation metrics" section that better clarifies our stated goal.

Comment 8: Also in the S3 material, it would be nice to see some illustrative examples of the PRC curves with key tests rather than just having the summary in box plots; something to justify the use of the summary statistics.

We understand that seeing illustrative examples of PRC curves could be useful. However, we do not include any as we have trained 1000s of machine learning classifiers, and being able to pick a "representative" PRC curve would be quite subjective, unlikely to give any extra insight into the GenePlexusZoo method, and might even be misleading (when calling something arbitrarily picked as 'representative'). Nevertheless, researchers will be able to use the released code to generate the PRC curves.

Comment 9: In the Results section, the first key result presented is under 'Multi-species network representations improve within-species gene classification'. I have a problem with the way these results are described compared to the accompanying Figure 2. The text describes features from six species performing significantly better than features from a single species. The word significant has a specific statistical meaning and should not be used here. Additionally, the BioGRID results do not show a strong result and the boxplots of log2(auPRC/prior) have largely overlapping quantiles across all categories. The only standout is the Human Only N2V with IMP for Function Annotation.

We appreciate the reviewer for carefully examining the figures and raising a fair concern. We have now added statistical tests comparing pairs of representations to each other. For Fig. 2 and 3, we consider each network–gene-set collection prediction task as a distinct task. We then

choose the single-species (the species the prediction task is for) adjacency matrix feature set as the baseline representation to which all other feature sets will be compared to. The log2(auPRC/prior) values for all gene sets within a gene set collection are compared using a Wilcoxon signed-rank test. Then, we perform Bonferroni correction for multiple testing and set an FWER threshold of 0.01 to declare a representation as being significantly different from baseline. For Fig. 4, a similar approach is taken except the baseline is the paired-species adjacency matrix representation.

We have added a new section in the Supplementary Material (**File S3**) titled "Statistical Tests for Main Results". In this section, we explain how we performed statistical testing. This section also includes figures for the statistical tests performed to summarize the results of **Figs. 2–4** and **Figs. SM10–12**. We now describe these results in the main manuscript in the **Results** section where these figures are first discussed.

Comment 10: Additionally, the scales should be the same (this goes for all comparison plots in both the main text and the S3 material) as it is not immediately apparent that the Phenotype IMP plot shows much worse results than the other three.

We agree that, as a general principle, keeping the same scale makes data across plots more easily/fairly comparable. However, in our case, we do not believe that the figure panels in question should be on the same scale for the following reasons. The goal of this study is to find out how the different feature sets impact performance in a given task. Therefore, the comparisons under focus are *within* each panel and making each panel have a scale that captures the breadth of performance allows for clearer comparison between feature sets visualized in that panel. Further, different prediction tasks have different levels of difficulty; for instance, phenotype prediction is much harder than function prediction. The choice of network can also greatly affect performance. We note that, in our original GenePlexus paper (Liu et al., 2020), we displayed the results in the same way (all panels having their own scale) for the same reasons given above. To better clarify the goal of our analysis, we have now added a sentence to the end of the first paragraph in the "Multi-species network representations improve within-species gene classification" section.

Comment 11: In figure 3 I have similar issues with the BioGRID results. The IMP results for Functional Annotation also show AdjMat (model) being close to or better than other methods for different organisms. There are some graphical artifacts with some of the box plots on the right side (also throughout S3) that should be fixed. The phenotype with IMP results has AdjMat stand out with yeast and fly.

For a part of this comment, kindly see our response to Comment 9 above about how statistical tests have been added to provide more clarity to these results.

Next, by "graphical artifacts", we believe that the reviewer is referring to cases where a boxplot with a notch has a "flipped" appearance. This appearance means that the 1st quartile has a lower value than the lower value of the confidence interval around the median (and similarly, the

3rd quartile is higher than the higher value). Although this may look like a graphical artifact, it is a reflection of the underlying data distribution and is actually useful information about the confidence in the median (or the lack thereof). A useful post with corresponding link to a github issue can be found at https://stackoverflow.com/questions/26291082/weird-behavior-of-matplotlibs-boxplot-when-using-the-notch-shape.

Comment 12: The second section, 'Multi-species network embedding enable cross-species gene classification' shows the capabilities of transfer learning. Figure 4 agrees with the description in the text and shows a noticeable but not major increase in log2(auPRC/prior).

Kindly see our response to Comment 9 above about how statistical tests have been added to provide more clarity on these results.

Comment 13: The last result, 'Mapping human diseases across species using all-species network embeddings' shows a practical exploration of Bardet-Biedl Syndrome 1 (BBS) as a demonstration of GenePlexusZoo's potential application. This case study approach, where abstract methods are applied to concrete problems, effectively communicates the tool's real-world significance.

We appreciate the comment.

Comment 14: The Discussion section is well written and addresses many of the particular elements with Figure 3 and Figure 4 from the Results section that I talked about earlier. It also presents the core claims of the study again which I feel were reasonably supported by the results. The significance of these claims lies in the potential of the framework to bridge the knowledge gap between species and enhance the predictive power in gene classification. This approach is innovative, with its strength lying in consolidating information from multiple species. The authors seem self-aware of the strengths and limitations of the methods. They point out that the novelty lies in the framework and not the exact method of generating the feature space, suggesting room for future research. However, while they mention that there are multiple ways to represent networks, a clearer emphasis on the challenges and limitations in creating these representations would add depth to the discussion.

We thank the Reviewer and address their comment on adding a clearer emphasis on the challenges and limitations in creating these representations by now adding a paragraph to **Discussion** section that describes the 4 main challenges in generating these feature spaces; 1) the ability to handle many-many orthologs, 2) the large number of nodes as the number of species is increased, 3) the method that dictates how to connect the edges across species, and 4) the computational resources needed to generate the feature spaces.

Comment 15: I briefly reviewed the code on GitHub and found it was very well written and organized. The supporting information was well put together and the figures for the main text were appropriately selected.

We thank the Reviewer for taking the time to read over our code base.

Comment 16: Overall I found that the manuscript was well written but several things need to be addressed. The comparative plots should have the same scale on the log2(auPRC/prior) axis in my opinion. It is the same metric and presumably, we should be evaluating the performance of each comparison using a common scale. Some of the wording on the results seems too strong compared to the figures and I think a more careful treatment of the results and claims would be appropriate.

We thank the Reviewer for their careful reading of the manuscript. We believe we have addressed all their points through the responses to all the other comments above.

Response To Reviewer 3:

Comment 1: The article presents an idea of leveraging genetic connections between species to aid in network-based gene classification tasks. The idea is interesting, and the proposed framework seems to produce better results than using information from the target species only. This might be due to incompleteness of information on any single species or other factors. The article is fairly well written, and can be accepted for publication with minor revision.

We thank the Reviewer for their recommendation about our work.

Comment 2: In the abstract (and later on p. 2 in the introduction), it is said that “some recent methods […] using networks from more than one species, they lack [the property of] handling networks from multiple species”. This sounds odd and somewhat contradictory.

We see how this could be confusing. The point we are trying to make is that almost all previous methods are restricted to using networks from two species at a time, whereas GenePlexusZoo can incorporate network information from >2 species seamlessly. In this work, we simultaneously use networks from six different species, and the method should be able to scale to even more. We have changed relevant text in the **Abstract** and in the 3rd paragraph of the **Introduction** section.

Comment 3: p. 2, second paragraph: says “human to model species to human, or vice versa”. The sentence immediately following, has “most current methods” and “often”. Maybe one should be deleted.

These grammatically incorrect statements have been changed by deleting the phrase “to human” in the first sentence and “often” in the second sentence.

Comment 4: In my opinion, the bullet points in the beginning of the “Results” section as well as Figure 1 could fit better in the introduction (the final choice is of course the authors’).

We have added a reference to **Figure 1** in the last paragraph of the "Introduction" section. If accepted, during the final editing of the manuscript, we will move **Figure 1** closer to the introduction as well.

We have decided to leave the bullet points at the start of the **Results** section. Similar text is already at the end of the **Introduction** section, and we would like to reinforce to readers very clearly a second time the main objectives the results will try to achieve.

Comment 5: The hyperparameter selection results are completely left to supplementary material. Perhaps some summary could be included in the main text. Moreover, it seemed that these results were not really presented in their entirety. That is, the effect of different hyperparameter selections are not visualised anywhere. It is also not clear, how the N2Vselected parameters are chosen. Is that the overall best paramteter combination out of those tested?

We have added a few sentences to the second paragraph of "Creating joint multi-species network representations using GenePlexusZoo" section describing the hyperparameter tuning steps. These methods are also now mentioned in the **Methods** section in response to Reviewer 2's Comment 2.

We now include the hyperparameter tuning results as part of the public github repository associated with this manuscript. These notebooks provided extensive plots for hyperparameter tuning across all the different tasks in which it was performed. Additional text was added to the "Optimal choices for gene classification using multi-species networks" section of the Supplemental Materials (**File S3**) to alert readers to this. In that section we also now list more of the hyperparameters chosen for the N2V method.

For selecting the best N2V parameter, the first choice was to select one set of parameters for every prediction task (i.e. network–gene-set-collection combination). This was done as it was not possible (due to computational resources) to do the full hyperparameter tuning for anything but the human and mouse combination. To select this set of parameters, we looked across all hyperparameter results from the human and mouse analyses. We note here that the goal of this work was to see if adding information from multiple species increased performance of over using the adjacency matrix from one species (i.e. the best performing representation from the original GenePlexus work).

Comment 6: In the section "Multi-species network embeddings enable cross-species gene classification" in the results, it is really difficult to understand what kind of data is used as training and ground truth data. Moreover, the results include "Ranks of matching terms", but they could be explained better. At least I cannot figure out how these results are obtained.

We understand how this is confusing and not properly explained. The idea here is that we developed a matched set of GO terms (see "Matched non-redundant gene sets across species" section in the "Methods" section) where, for each species pair, we only include a GO term if it

had enough annotations in both species to train a machine learning classifier. From here, we then train a classifier on the human annotations and predict on the model species annotations. We then can find the log2(auPRC/prior) for every gene set in the matched gene set collection using only model species annotations. In the top row of Figure 4, we just display the log2(aupRC/prior) of the GO term for which the classifier was trained, giving an idea on the absolute performance of the classifier to predict model species gene annotations annotated to the same GO term. The bottom row of Figure 4 is a relative comparison in which we ranked the log2(auPRC/prior) of the ML classifier that uses model species gene annotations annotated to the same GO term versus all other GO terms in the gene set collection. The idea here is that a matched gene set should have the highest log2(auPRC/prior) compared to all the other gene sets in the collection (i.e. a ranking of 1). We have added text to the “Multi-species network embeddings enable cross-species gene classification” section in the **Results** section to better explain this.
