# Supplementary material for: Joint representation of molecular networks from multiple species improves gene classification
Source: PLoS Comput Biol. 2024 Jan 10;20(1):e1011773. doi: 10.1371/journal.pcbi.1011773 (PMC10805316; doi:10.1371/journal.pcbi.1011773)
Supplement: Attachment — Submitted filename: GenePlexusZoo_PLoSCB-Response-to-Reviewers_v3.pdf [file pcbi.1011773.s006.pdf]

Response to the Reviewers for PCOMPBIOL-D-23-01096
We would like to thank the reviewers for carefully reading our manuscript and for their helpful comments. We have provided detailed responses to all the comments below.

In the following, **Reviewer comments** are in **black**, **our responses** are in **blue**, and the **changes made to the text of our paper** are shown in **red**.

Response To Reviewer 3:
Comment 1: I am happy with the revised manuscript and the authors' responses. The manuscript can be accepted as such.

We thank the reviewer for the recommendation.

Response To Reviewer 4:
Comment 1: In this paper, the authors propose GenePlexusZoo, a framework to cast molecular networks from multiple species into a single reusable feature space and this framework improves the gene classification tasks. Although the paper is overall clearly written, we have several major concerns about the methodology. In our opinion, these concerns, if not addressed, will preclude GenePlexusZoo to be a useful data analysis tool.

We thank the reviewer for their careful reading of our work.

Comment 2: In section “introduction”, the authors mentioned that “Most current methods for transferring genetic and molecular knowledge across species are restricted to using one-to-one orthologs, and not those with one-to-many and many-to-many relationships”. However, a recent published paper, “Cross-species cell-type assignment from single-cell RNA-seq data by a heterogeneous graph neural network” which modeled many-to-many and one-to-many orthologs across species to do cross--species cell-type assignment and cross-species gene module extraction tasks. I think this should be also accessed in introduction or discussion.

We have now included a brief description of this work in the Introduction section as it is an interesting application of cross species knowledge transfer using single cell data. We do note that in this work the Domainoid orthology method they used only includes pairs of proteins, and while orthology information included on the BioMart web server does include some many-to-many mappings, the scale in which the eggNOG database groups genes/proteins across species is much greater. Additionally, this work is only designed for pairs of species.

Comment 3: In section “Connecting networks across species”, the authors used orthology information from the eggNOG database, we are interested that whether change the orthology information e.g., using orthology information from BioMart, will influence the results. The impact of such disparity should be assessed and evaluated seriously.

We agree that the choice of the orthology database may impact the results. During the development of this project we compared two orthology methods: eggNOG and WORMHOLE (a

combination of several different orthology methods including eggNOG and bioMart/Ensembl). We now provide two supplemental figures showing results based on these two databases (Figs. SM-10-11) and mention this analysis in the Results section. It can be seen that, invariably, eggNOG has better or equivalent performance across most tasks. The overall comparable performance indicates cross-species gene links at genome-scale across multiple species obtained from a reasonable orthology inference method provides enough between-species gene relationship information to improve gene classification for single species annotations as well as transferring knowledge across species. This does not mean that any one particular orthology method might result in an increase or decrease in performance of some subset of classification tasks, but overall, we do not believe that different orthology methods (among well-benchmarked approaches; https://questfororthologs.org/) will dramatically and systematically impact network-based knowledge transfer.

Comment 4: In section "Evaluation scheme" the authors provided a study-bias holdout scheme that is close to the real-world situation, however the results of cross validation did not be included. The time and memory consumption of the computations should, at least be reported to demonstrate the reasonability of not conducting cross-validation. The computing platform also needs to be detailed.

We have added the higher-end computational requirements of models to the section "Evaluation scheme". The highest resources come from models in which the features are the influence matrix representations between species pairs. Here, models can have over 80k features, and thus the logistic regression algorithms need significant resources (500 GB RAM and over 24 hours) to train models in a single gene set collection. Coupled to these high computational demands for certain feature sets is the fact that we ran this on Michigan State University's high performance computing cluster. This is a heterogeneous collection of hardware added over more than a decade, thus with high variability in processing power and available memory. Although we have purchased some buy-in nodes on this system, due to the large number of models trained in this work, we relied heavily on general use nodes, and thus a major limiting factor is time jobs spend in the queue waiting to start. We now mention this high performance computing resource in the Acknowledgments section.

Comment 5: In Fig 2 and Fig 3, the authors reported the performance of predicting function- and phenotype-associated human genes using human, human + model, and all-species network representations. It seems that in the Function Annotation tasks, N2V (all-species) outperforms others in BioGRID and IMP networks. However, in Phenotype Annotation tasks, the AdjMat (human) method, which used Human only information achieved superior performance. So, what is the advantages of N2V (all-species) in this case? This, at the minimal, should be carefully evaluated.

To clarify, Figure 2 displays results for human gene annotation prediction, whereas Figure 3 displays results for model species gene annotation prediction.

For human gene annotation prediction the baseline method [AdjMAt (human)] never outperforms the all species embedding [N2V (all-species)]. In fact the baseline method is significantly outperformed by the all species embedding in Function prediction for both BioGRID and IMP and there is no statistical difference between the two methods for phenotype prediction in both BioGRID and IMP.

For model species gene annotation, in the function prediction task, similar results are seen to those for human gene function annotation. For phenotype gene annotation prediction in BioGRID there is no statistical difference between AdjMat (model) and NV2 (all-species) for any species except for yeast. This is similar to human phenotype gene annotation prediction where no statistically significant differences were seen between the all-species embedding method and the baseline.

For model species phenotype gene annotation prediction in IMP, the baseline method does outperform the all-species embedding for all species. We have changed the text in the Discussion section where we previously discussed why the single species adjacency matrix might be performing well in these cases and why using multi-species features, including embeddings, may still be important for these tasks to be more clear.

Comment 6: In section "Mapping human diseases across species using all-species network embeddings", the authors mentioned that "we observed that the top-ranked genes [Table SM5 in File S3], biological processes [Table SM6 in File S3], and phenotypes in the model species are relevant to cilium function and manifestations of the BBS disorder such as rod/cone dystrophy, polydactyly, and hypogonadism [Tables SM7 in File S3]". Also, in the Fig 5, the authors highlight the biological processes, e.g., microtubule-based movement, spindle elongation, axoneme assembly, the authors should, at the minimal, cite some literatures to validate these biological processes are relevant to cilium function and manifestations of the BBS disorder.

We believe that in the section "Mapping human diseases across species using all-species network embeddings" the Forsythe and Beales, 2013 citation is sufficient for the reader to understand the presentation of BBS as well as some of the mechanisms underlying the disease.

Although not enough research has been done on this rare disorder to define the exact perturbation of the individual biological processes, many of the processes in Figure 5 and Table SM6 are directly related to cilium function/development based on their definitions.

Secondly, our claim is not that the highlighted (bolded) processes in Figure 5 that are being referred to in this comment ("e.g., microtubule-based movement, spindle elongation, axoneme assembly", etc) are more relevant to BBS than the other processes in the figure. Rather, the processes in bold represent biological processes where, in at least one species, none of the annotated genes are orthologous to any human BBS gene. We have added wording to the figure caption to make this clearer.

We have added expanded text in the Discussion section to help make the relationships of the GO terms to BBS more clear.

Comment 7: In section “Gene set collections for different prediction tasks”, the authors mentioned that “we filtered the genes to retain only those that were found in a given network and filtered the terms to only those that had between a specified minimum and maximum number of annotations.” The minimum and maximum number of specified annotations should be explicitly specified.

This is included in Table SM2-4 in File S3. This has been noted in the section “Gene set collections for different prediction tasks”.

Comment 8: In Fig 4, the legend of three colors should be added.

We thank the reviewer for catching this as the legend was cropped off upon resubmission. We have reuploaded Figure 4 and Figures SM10-12.
