# Supplementary material for: Joint representation of molecular networks from multiple species improves gene classification
Source: PLoS Comput Biol. 2024 Jan 10;20(1):e1011773. doi: 10.1371/journal.pcbi.1011773 (PMC10805316; doi:10.1371/journal.pcbi.1011773)
Supplement: Attachment — Submitted filename: GenePlexusZoo_PLoSCB-Response-to-Reviewers_v4.pdf [file pcbi.1011773.s008.pdf]

Response to the Reviewers for PCOMPBIOL-D-23-01096R2
We would like to thank the reviewer for carefully reading our manuscript and for their helpful comments. We have provided detailed responses to all the comments below.

In the following, **Reviewer comments** are in **black**, **our responses** are in **blue**, and the **changes made to the text of our paper** are shown in **red**.

Response To Reviewer 4:

Comment 1: In section "introduction", the authors mentioned that "The CAME method integrates single-cell expression data across pairs of species using graph neural networks to improve cell-type assignment and discover shared cell-type-specific function in homologous gene modules (X. Liu et al., 2023). All of the above mentioned methods lack one or more desirable properties: 1) the ability to handle networks from more than two species simultaneously, 2) incorporating many-to-many orthology information, or 3) generating a representation that is reusable across many tasks (i.e., pathway, disease or phenotype prediction)". However, the statement in (2) may be misleading to readers since the CAME method do incorporate many-to-many orthology information,

We state very clearly that "the above mentioned methods lack **one** or more desirable properties" (i.e. the methods don't satisfy all three properties). The CAME method has only been demonstrated on pairs of species, thus does not satisfy property 1. Therefore, the language that we have used would not be misleading to readers.

Comment 2: In section "Evaluation scheme" the authors provided a study-bias holdout scheme that is close to the real-world situation, however the results of cross validation did not be included. In the revised version, the authors mentioned that "Here, models can have over 80k features, and thus the logistic regression algorithms need significant resources (500 GB RAM and over 24hours) to train models in a single gene set collection". The running time should be accurate, i.e. 25hours or 48 hours, to let the reader make an accurate judgment.

We believe that the current phrasing is sufficient to have a reader understand that enormous computational resources were required to complete the study in its present form, and these computational requirements prevent us from carrying out cross-validation.

Further, "runtime" cannot be easily reduced to a single number. Thousands of machine learning models were trained on datasets with various features dimensions, data sparsity, amount of labeled data, etc., on a collection of heterogeneous hardware (979 unique computational nodes), which can have vastly different amounts of processing power. Additionally, a major limitation is the queue length (i.e., how long it takes a job to start on the high performance computing system).

Lastly, evaluation based on cross-validation (CV using random splits of data) leads to overestimation performance and it is exactly for this reason that we show results from study bias

holdout, which more accurately reflects the real world problem of learning from well-studied genes and predicting on genes with limited or no prior knowledge. This was the main evaluation metric used in our previously published work on the subject where we show both CV and study-bias holdout. The Reviewer has not provided any reason in this review or their previous review as to the benefit cross-validation would bring to this work.

Comment 3: In section "Mapping human diseases across species using all-species network embeddings", the authors mentioned that "we observed that the top-ranked genes [Table SM5 in File S3], biological processes [Table SM6 in File S3], and phenotypes in the model species are relevant to cilium function and manifestations of the BBS disorder such as rod/cone dystrophy, polydactyly, and hypogonadism". In the rebuttal letter, the authors mentioned that "our claim is not that the highlighted (bolded) processes in Figure 5 that are being referred to in this comment ("e.g., microtubule-based movement, spindle elongation, axoneme assembly", etc) are more relevant to BBS than the other processes in the figure. Rather, the processes in bold represent biological processes where, in at least one species, none of the annotated genes are orthologous to any human BBS gene". I wondered, since these the processes in bold represent biological processes where, in at least one species, none of the annotated genes are orthologous to any human BBS gene, how to verify the relevance of these processes in the context of BBS? Since the title of figure 5 is "The ten most enriched biological processes associated with the top genes in each species predicted to be related to Bardet-Biedl Syndrome 1 (BBS)", I believe it is necessary to verify the relevance of these processes to BBS.

We believe this concern has been addressed in the previous revision with the following text:

*As evidence of this capability, GenePlexusZoo picks out highly similar processes and phenotypes in model species even when they share no orthologs with BBS in human [Fig. 5]. A major mechanism underlying **BBS is disruption to the meditation and regulation of microtubule-based intracellular transport processes** (Forsythe and Beales, 2013; Blacque and Leroux, 2006; Zaghloul and Katsanis, 2009). A number of top predicted biological processes are microtubule-based intracellular transport processes (**microtubule-based movement, microtubule-based movement, transport along microtubule, axoneme assembly, organelle transport along microtubule**, etc.), however the model species genes annotated to those specific gene ontology terms have no known orthologs to the human BBS genes.*

Here, based on published literature, we clearly identify the major mechanism underlying BBS (bolded above) and verified that a number of processes in model species (bolded above) are directly related to this disease mechanism, where the genes annotated to these processes have no shared orthologs with the human gene annotations for BBS.

We note that, in this manuscript, it is clear that we are not claiming that every biological process predicted to be associated with BBS is in fact so. However, we are providing an illustrative example of how GenePlexusZoo can be used to discover meaningful insights across many species using a single network representation.
