# Supplementary material for: Joint representation of molecular networks from multiple species improves gene classification
Source: PLoS Comput Biol. 2024 Jan 10;20(1):e1011773. doi: 10.1371/journal.pcbi.1011773 (PMC10805316; doi:10.1371/journal.pcbi.1011773)
Supplement: Attachment — Submitted filename: Reviewer Attachments_231127.docx [file pcbi.1011773.s005.docx]

In this paper, the authors propose GenePlexusZoo, a framework to cast molecular networks from multiple species into a single reusable feature space and this framework improves the gene classification tasks. Although the paper is overall clearly written, we have several major concerns about the methodology. In our opinion, these concerns, if not addressed, will preclude GenePlexusZoo to be a useful data analysis tool.

Below please find our detailed comments:

1. In section “introduction”, the authors mentioned that “*Most current methods for transferring genetic and molecular knowledge across species are restricted to using one-to-one orthologs, and not those with one-to-many and many-to-many relationships*”. However, a recent published paper, “Cross-species cell-type assignment from single-cell RNA-seq data by a heterogeneous graph neural network” which modeled many-to-many and one-to-many orthologs across species to do cross--species cell-type assignment and cross-species gene module extraction tasks. I think this should be also accessed in introduction or discussion.
2. In section “Connecting networks across species”, the authors used orthology information from the eggNOG database, we are interested that whether change the orthology information e.g., using orthology information from BioMart, will influence the results. The impact of such disparity should be assessed and evaluated seriously.
3. In section “Evaluation scheme” the authors provided a study-bias holdout scheme that is close to the real-world situation, however the results of cross validation did not be included. The time and memory consumption of the computations should, at least be reported to demonstrate the reasonability of not conducting cross-validation. The computing platform also needs to be detailed.
4. In Fig 2 and Fig 3, the authors reported the performance of predicting function- and phenotype-associated human genes using human, human + model, and all-species network representations. It seems that in the Function Annotation tasks, N2V (all-species) outperforms others in BioGRID and IMP networks. However, in Phenotype Annotation tasks, the AdjMat (human) method, which used Human only information achieved superior performance. So, what is the advantages of N2V (all-species) in this case? This, at the minimal, should be carefully evaluated.
5. In section “Mapping human diseases across species using all-species network embeddings”, the authors mentioned that “*we observed that the top-ranked genes [Table SM5 in File S3], biological processes [Table SM6 in File S3], and phenotypes in the model species are relevant to cilium function and manifestations of the BBS disorder such as rod/cone dystrophy, polydactyly, and hypogonadism [Tables SM7 in File S3]*”. Also, in the Fig 5, the authors highlight the biological processes, e.g., microtubule-based movement, spindle elongation, axoneme assembly, the authors should, at the minimal, cite some literatures to validate these biological processes are relevant to cilium function and manifestations of the BBS disorder.
6. In section “Gene set collections for different prediction tasks”, the authors mentioned that “*we filtered the genes to retain only those that were found in a given network and filtered the terms to only those that had between a specified minimum and maximum number of annotations.*” The minimum and maximum number of specified annotations should be explicitly specified.
7. In Fig 4, the legend of three colors should be added.
