# Supplementary material for: Joint representation of molecular networks from multiple species improves gene classification
Source: PLoS Comput Biol. 2024 Jan 10;20(1):e1011773. doi: 10.1371/journal.pcbi.1011773 (PMC10805316; doi:10.1371/journal.pcbi.1011773)
Supplement: Attachment — Submitted filename: Reviewer Attachments_231205.docx [file pcbi.1011773.s007.docx]

I'd like to thank the authors for the updates and revisions. However, we still have some concerns about the authors’ claim in the rebuttal letter, below please find our detailed comments:

1. In section “introduction”, the authors mentioned that “*The CAME method integrates single-cell expression data across pairs of species using graph neural networks to improve cell-type assignment and discover shared cell-type-specific function in homologous gene modules (X. Liu et al., 2023). All of the above mentioned methods lack one or more desirable properties: 1) the ability to handle networks from more than two species simultaneously, 2) incorporating many-to-many orthology information, or 3) generating a representation that is reusable across many tasks (i.e., pathway, disease or phenotype prediction)*”. However, the statement in (2) may be misleading to readers since the CAME method do incorporate many-to-many orthology information,
2. In section “Evaluation scheme” the authors provided a study-bias holdout scheme that is close to the real-world situation, however the results of cross validation did not be included. In the revised version, the authors mentioned that “*Here, models can have over 80k features, and thus the logistic regression algorithms need significant resources (500 GB RAM and over 24hours) to train models in a single gene set collection*”. The running time should be accurate, i.e. 25hours or 48 hours, to let the reader make an accurate judgment.
3. In section “Mapping human diseases across species using all-species network

embeddings”, the authors mentioned that “*we observed that the top-ranked genes [Table SM5 in File S3], biological processes [Table SM6 in File S3], and phenotypes in the model species are relevant to cilium function and manifestations of the BBS disorder such as rod/cone dystrophy, polydactyly, and hypogonadism*”. In the rebuttal letter, the authors mentioned that “*our claim is not that the highlighted (bolded) processes in Figure 5 that are being referred to in this comment (“e.g., microtubule-based movement, spindle elongation, axoneme assembly”, etc) are more relevant to BBS than the other processes in the figure. Rather, the processes in bold represent biological processes where, in at least one species, none of the annotated genes are orthologous to any human BBS gene*”. I wondered, since these the processes in bold represent biological processes where, in at least one species, none of the annotated genes are orthologous to any human BBS gene, how to verify the relevance of these processes in the context of BBS? Since the title of figure 5 is “*The ten most enriched biological processes associated with the top genes in each species predicted to be related to Bardet-Biedl Syndrome 1 (BBS)*”, I believe it is necessary to verify the relevance of these processes to BBS.
